# A TRAF3-NIK module differentially regulates DNA vs RNA pathways in innate immune signaling

**DOI:** 10.1038/s41467-018-05168-7

**Published:** 2018-07-17

**Authors:** Kislay Parvatiyar, Jose Pindado, Anurupa Dev, Saba Roghiyh Aliyari, Shivam A. Zaver, Hoda Gerami, Maxime Chapon, Amir A. Ghaffari, Anant Dhingra, Genhong Cheng

**Affiliations:** 1 Department of Microbiology, Immunology, and Molecular Genetics, UCLA, Los Angeles, CA 90095 USA; 2 Molecular Biology Institute, University of California, Los Angeles (UCLA), Los Angeles, CA 90095 USA; 3 Medical Scientist Training Program, David Geffen School of Medicine UCLA, Los Angeles, CA 90095 USA; 4 Jonsson Comprehensive Cancer Center, UCLA, Los Angeles, CA 90095 USA

## Abstract

Detection of viral genomes by the innate immune system elicits an antiviral gene program mediated by type I interferons (IFNs). While viral RNA and DNA species induce IFN via separate pathways, the mechanisms by which these pathways are differentially modulated are unknown. Here we show that the positive regulator of IFN in the RNA pathway, TRAF3, has an inhibitory function in the DNA pathway. Loss of TRAF3 coincides with increased expression of the alternative NF-κB-inducing molecule, NIK, which interacts with the DNA pathway adaptor, STING, to enhance IFN induction. Cells lacking NIK display defective IFN activation in the DNA pathway due to impaired STING signaling, and NIK-deficient mice are more susceptible to DNA virus infection. Mechanistically, NIK operates independently from alternative NF-κB signaling components and instead requires autophosphorylation and oligomerization to activate STING. Thus a previously undescribed pathway for NIK exists in activating IFN in the DNA pathway.

## Abstract (web summary)

The innate immunity system detects viral pathogens by sensing viral DNA or RNA via distinct pathways, but whether these pathways cross-regulate is unclear. Here the authors show that TRAF3, a known regulator of the RNA-sensing pathway, modulates an NF-κB activator NIK to control DNA-sensing by the adaptor STING in immune cells.

## Introduction

Innate immunity provides a critical first step in initiating host defense against microbial pathogens. In the innate immune response to viral infections, the induction of type I interferon (IFN) cytokines, including IFN-β/-α, is fundamental as they elicit the potent activation of an antiviral cellular gene program that rapidly culminates in the inhibition of viral replication and spread^1,2^.

Activation of IFN is regulated primarily by the interferon regulatory factor 3 (IRF3) transcription factor that undergoes phosphorylation-dependent activation by the upstream TANK-binding kinase 1 (TBK1). Innate immune activation of the TBK1/IRF3 signaling axis is largely governed by engagement of select pattern recognition receptors (PRRs) that recognize viral genomes. Certain membrane-bound PRRs can detect viral nucleic acids on the cell surface or in endosomes while intracellular PRRs detect viral nucleic acids confined to cytosolic compartments^2,3^. These cytosolic PRRs can be categorized by the nucleic acid types they detect: RNA species, particularly double-stranded RNA (dsRNA) is sensed by the retinoic acid inducible gene I (RIG-I, aka DDX58) or melanoma differentiation associated gene 5 (MDA5) helicases, whereas dsDNA species are sensed by cyclic guanosine adenosine monophosphate synthase (cGAS, aka MB21D1), DEAD-box helicase 41 (DDX41), or interferon gamma-inducible protein 16 (IFI16). In the dsRNA-sensing PRR pathway, signaling to TBK1/IRF3 to activate IFN requires the mitochondrial adaptor, interferon-β promoter stimulator-1 (IPS-1, aka MAVS/CARDIF/VISA) (RNA/IPS-1 pathway). Alternatively, in the dsDNA-sensing PRR pathway, activation of IFN via TBK1/IRF3 is conferred via the endoplasmic reticulum adaptor, stimulator of interferon genes (STING, aka TMEM173/MPYS/MITA/ERIS) (DNA/STING pathway)^2,4–6^.

Although extensive studies have focused on the activation and regulation of the RNA or the DNA pathways, our understanding of how they are differentially regulated are poorly understood while the mechanisms that govern how these pathways crosstalk with each other is unknown. Work by our group and others previously defined a critical role for tumor necrosis factor (TNF) receptor-associated factor 3 (TRAF3) in mediating the activation of IFN in the RNA pathway. Mechanistically, TRAF3 associated with the RNA pathway adaptor, IPS-1, to drive signal activation to the TBK1–IRF3 signaling axis^7–10^. However, the role of TRAF3 in the DNA pathway has not been determined. Here we demonstrate that TRAF3 plays a direct opposite role in the DNA pathway, functioning as a negative regulator of DNA virus or DNA species-mediated activation of the IFN response. TRAF3 licensed the suppression of the alternative nuclear factor (NF)-κB inducing kinase (NIK, aka MAP3K14), which resulted in impaired IFN activation in the DNA pathway while displaying a heightened IFN response in the RNA pathway. NIK associated with the DNA pathway adaptor, STING, to enhance its activation via an alternative NF-κB pathway-independent mechanism. Together, our data describe a unique signaling module (TRAF3/NIK) that oppositely regulates the same signaling output (IFN) in a manner dependent on the signaling input (nucleic acid type), ultimately revealing a novel regulatory crosstalk mechanism between the RNA and the DNA pathway.

## Results

### TRAF3 plays opposing roles in regulating IFN in the RNA vs DNA pathways

**Fig. 1:**
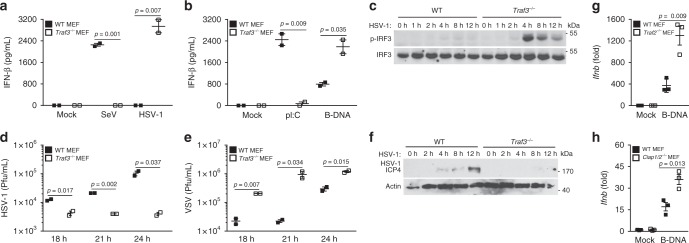
The modulators of the alternative NF-κB pathway play a negative regulatory role in the DNA pathway. **a**, **b** IFN-β quantification via ELISA from supernatants collected 24 h postinfection or stimulation of WT and *Traf3*^*−/−*^ MEFs infected with Sendai virus (SeV) or herpes simplex virus 1 (HSV-1) (MOI 0.1) (**a**) or transfected with poly I:C (100 ng/mL) or B-DNA (500 ng/mL) (**b**). Data are means ± SEM of one experiment run in duplicates out of 2–3 independent experiments. **c** Immunoblot analysis of IRF3 activation in WT and *Traf3*^*−/−*^ MEF cells infected HSV-1 (MOI 0.1) at the indicated time points. Results are representative of two independent experiments. **d**, **e** Viral titers determined via plaque assay from supernatants of WT and *Traf3*^*−/−*^ MEFs infected with HSV-1 (MOI 0.1) (**d**) or vesicular stomatitis virus (VSV) (MOI 0.01) (**e**) analyzed 18, 21, and 24 h postinfection. Data are means ± SEM of one experiment run in duplicates out of 2–3 independent experiments. **f** Immunoblot analysis of HSV-1 encoded protein ICP4 in WT and *Traf3*^*−/−*^ MEF cells infected HSV-1 (MOI 0.1) at the indicated time points. Actin serves as a loading control. Results are representative of two independent experiments. **g**, **h** Gene expression levels of IFN-β determined via Q-PCR in WT and *Traf2*^*−/−*^ MEFs (**g**) or in WT and *ciap1/2*^*−/−*^ MEFs (**h**) stimulated with B-DNA (500 ng/mL) for 4 h. Data are means ± SEM of one experiment run in triplicates out of 2–3 independent experiments. *p* < 0.05 is considered significant

TRAF3 functions as a key positive regulator of IFN in the RNA pathway. We sought to determine whether TRAF3 was also critical in activating IFN in the DNA pathway. As expected, murine embryonic fibroblasts (MEFs) lacking TRAF3 displayed impaired IFN (IFN-β or IFN-α cytokine) production when infected with the RNA virus, Sendai virus (SeV), or transfected with the dsRNA mimetic, poly I:C (pI:C), when compared to wild-type (WT) control MEFs. Surprisingly, *Traf3*^*−/−*^ MEFs infected with the DNA virus, herpes simplex virus 1 (HSV-1), or transfected with dsDNA, poly dA:dT (B-DNA) or other DNA species, presented a polar opposite response, showcasing elevated levels of IFN compared to the WT MEFs (Fig. 1a, b and Supplementary Figure 1A–B).

IFN activation is primarily dependent on the IRF3 transcription factor that requires phosphorylation-dependent activation by the upstream kinase, TBK1. *Traf3*^*−/−*^ MEFs infected with HSV-1 displayed increased levels of TBK1 and IRF3 phosphorylations, whereas *Traf3*^*−/−*^ MEFs infected with SeV displayed reduced TBK1 and IRF3 phosphorylations when compared to WT MEFs (Fig. 1c and Supplementary Figure 1C).

In innate antiviral immune pathways, IFN activates a Janus-activated kinase-signal transducer and activator of transcription factor (STAT) signaling cascade to initiate the induction of an antiviral state. *Traf3*^*−/−*^ MEFs infected with HSV-1 displayed increased levels of STAT1 phosphorylation compared to WT MEFs (Supplementary Figure 1D). Consequently, TRAF3-deficient MEFs presented with reduced DNA viral loads of HSV-1 and the murine herpes virus 68 (MHV-68), which was in contrast to TRAF3-deficient MEFs that presented with increased RNA viral loads of vesicular stomatitis virus (VSV) in comparison to WT MEFs (Fig. 1d, e and Supplementary Figure 1E). Immunoblotting analysis further showed dampened expression of DNA virus-encoded proteins in *Traf3*^*−/−*^ MEFs infected with HSV-1 (Fig. 1f and Supplementary Figure 1F).

To confirm the DNA pathway-induced levels of the heightened IFN response displayed in the *Traf3*^*−/−*^ MEFs was indeed due to TRAF3, *Traf3*^*−/−*^ MEFs were reconstituted with either empty vector or a plasmid encoding TRAF3. IFN levels were then monitored after DNA virus infection. As expected, *Traf3*^*−/−*^ MEFs harboring empty vector plasmid showed elevated IFN levels after HSV-1 infection compared to WT MEFs. However, *Traf3*^*−/−*^ MEFs transduced with the TRAF3 plasmid showed decreased IFN expression when infected with HSV-1 (Supplementary Figure 1G–H). As such, *Traf3*^*−/−*^ MEFs reconstituted with TRAF3 displayed viral loads comparable to WT MEFs when infected with HSV-1 (Supplementary Figure 1I). To further confirm that TRAF3 functioned as a negative regulator of the DNA pathway, the DNA-induced IFN response was examined in bone marrow-derived macrophages (BMDMs) derived from TRAF3-deficient mice. Similar to *Traf3*^*−/−*^ MEFs, *Traf3*^*−/−*^ BMDMs infected with HSV-1 also displayed elevated levels of IFN when compared to BMDMs derived from WT mice (Supplementary Figure 1J–K).

TRAF3 functions as a key negative regulator of an alternative NF-κB signaling pathway where it operates as part of a multiprotein complex containing TRAF2 and cellular inhibitor of apoptosis 1 and 2 (cIAP1 and cIAP2, aka BIRC2 and BIRC3)^11,12^. We therefore examined whether these other inhibitory components phenocopied TRAF3 in inhibiting the DNA pathway. *Traf2*^*−/−*^ MEFs showed impaired IFN production in response to SeV infection or pI:C transfection (Supplementary Figure 1L–M) compared to WT MEFs. In contrast, B-DNA transfected or HSV-1-infected *Traf2*^*−/−*^ MEFs displayed a heightened IFN response, similar to that of *Traf3*^*−/−*^ MEFs (Fig. 1g and Supplementary Figure 1N) and consequently showed increased levels of STAT1 phosphorylation upon DNA virus infection (Supplementary Figure 1O). Similar to TRAF3, cIAP1 and cIAP2 have been demonstrated to function as positive regulators of the RNA pathway^13^. Consistent with the TRAF3 and TRAF2 phenotype observed in the DNA pathway, cIAP1/2 doubly deficient MEFs displayed increased IFN activation when transfected with B-DNA (Fig. 1h and Supplementary Figure 1P).

These results uncover a surprising role for TRAF3 and its alternative NF-κB inhibitory multiprotein complex constituents, TRAF2, cIAP1, and cIAP2, in positively regulating IFN in the RNA pathway while negatively regulating IFN in the DNA pathway.

### NIK is a positive regulator of the DNA pathway

**Fig. 2:**
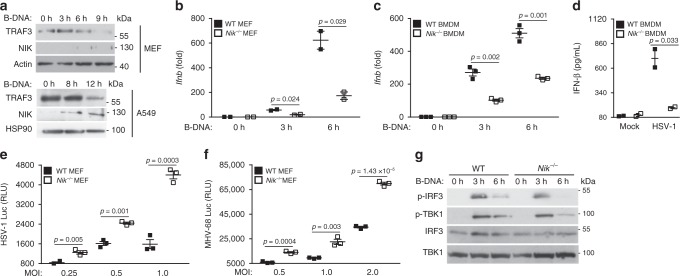
NIK is a positive regulator of the DNA pathway. **a** Immunoblot analysis of TRAF3 and NIK in MEF cells (upper panel) or A549 cells (lower panel) transfected with B-DNA (2 μg/mL) for the indicated time points. Actin and HSP90 serve as loading controls. Results are representative of three independent experiments. **b**, **c** IFN-β mRNA levels determined via Q-PCR in WT and *Nik*^*−/−*^ MEFs (**b**) transfected with B-DNA (500 ng/mL) or in WT and *Nik*^*−/−*^ BMDMs (**c**) transfected with B-DNA (100 ng/mL) for the indicated time points. Data are means ± SEM of one experiment run in duplicates (**b**) or triplicates (**c**) out of 2–3 independent experiments. **d** ELISA for IFN-β in supernatants collected 24 h after infection from WT and *Nik*^*−/−*^ BMDMs infected with HSV-1 (MOI 5.0). Data are means ± SEM of one experiment run in duplicates out of two independent experiments. **e**, **f** Viral loads of HSV-1 encoding luciferase (**e**) or murine herpes virus 68 (MHV-68) encoding luciferase (**f**) in WT and *Nik*^*−/−*^ MEFs infected with virus at the indicated MOIs. Data are means ± SEM of one experiment run in triplicates out of two independent experiments. **g** Immunoblot analysis for IRF3 and TBK1 activation in WT and *Nik*^*−/−*^ BMDMs transfected with B-DNA (1 μg/mL) for the indicated time points. Results are representative of two independent experiments. *p* < 0.05 is considered significant

In most resting cells, TRAF3, TRAF2, cIAP1, and cIAP2 expression is static as they cooperate to suppress NIK, a key instigator of alternative NF-κB signaling^14–16^. Indeed, cells lacking any of these inhibitory components have been shown to display an increase in NIK protein expression. To determine the physiological relevance of TRAF3 in the DNA pathway, cells were transduced with B-DNA. Immunoblot analysis revealed that DNA pathway activation caused a decrease in TRAF3 levels. Furthermore, the loss of TRAF3 expression was concomitant with an increase in NIK expression in B-DNA-transduced cells (Fig. 2a). We therefore hypothesized that the increased levels of IFN presented in the cells lacking TRAF3 when infected with DNA virus or transfected with B-DNA were imparted by NIK. To test this, *Traf3*^*−/−*^ MEFs were transfected with small interfering RNA (siRNA) targeting NIK. Indeed, B-DNA-mediated activation of IFN in TRAF3-deficient MEFs was dampened when NIK was knocked down (Supplementary Figure 2A).

To verify a role for NIK in facilitating the enhanced IFN activation phenotype in response to the DNA pathway, cells lacking NIK were transfected with B-DNA and monitored for IFN activation. Indeed, NIK-deficient MEFs and BMDMs displayed defective induction of IFN in response to B-DNA and other DNA species (Fig. 2b, c and Supplementary Figure 2B–C). BMDMs lacking NIK also showed impaired IFN induction in response to infections with HSV-1 and MHV-68, as well as with the intracellular bacterium, *Listeria monocytogenes*, known to activate IFN via the DNA pathway (Fig. 2d and Supplementary Figure 2D)^17,18^. As such, the defective IFN responses in the NIK-deficient cells resulted in higher DNA viral loads compared to infected WT cells (Fig. 2e, f). In contrast, and consistent with published reports^19^, NIK played an opposite role in the RNA pathway, functioning as a negative regulator of RNA-mediated activation of IFN as NIK-deficient cells harbored reduced RNA viral loads compared to WT control cells (Supplementary Figure 2E–F).

To determine whether the impaired IFN activation presented in the NIK-deficient cells in response to the DNA pathway was due to dysfunctional IRF3 activation, immunoblot analysis was performed. Indeed, NIK-deficient BMDMs and MEFs displayed reduced IRF3 phosphorylation in response to B-DNA stimulation when compared to WT control cells (Fig. 2g and Supplementary Figure 2G–H). Notably, cells lacking NIK showed no obvious reduction in expression of key upstream signaling components specific to the DNA pathway (Supplementary Figure 2I)^20,21^.

To confirm that the impaired IFN induction in the DNA pathway as well as the heightened DNA viral levels displayed in the NIK-deficient cells were in fact imparted by NIK, *Nik*^*−/−*^ MEFs were reconstituted with either empty vector or a plasmid-expressing NIK followed by transfecting or infecting the cells with B-DNA or HSV-1, respectively. Indeed, cells lacking NIK that were reconstituted with NIK showed an increase in IFN activation in response to B-DNA stimulation and harbored lower levels of HSV-1 when compared to NIK-deficient cells transduced with a control empty vector (Supplementary Figure 2J–L).

Taken together, our data indicate that the TRAF3-regulated protein, NIK, functions as a positive regulator of the DNA pathway while serving an opposite role in the RNA pathway. Thus a TRAF3/NIK module differentially controls RNA vs DNA pathway activation in innate antiviral signaling to IFN.

### NIK enhances STING signaling

**Fig. 3:**
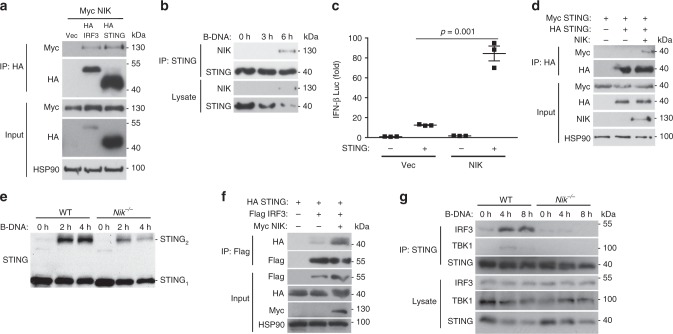
NIK enhances STING activation in the DNA pathway. **a** Co-immunoprecipitation and immunoblot of HA-IRF3 or HA-STING with Myc-NIK co-transfected in HEK 293T cells. Results are representative of three independent experiments. **b** Immunoblot analysis of STING–NIK interactions in MEF cells transfected with B-DNA (2 μg/mL) for the time points indicated and immunoprecipitated for STING. Results are representative of two independent experiments. **c** IFN-β luciferase reporter assay in HEK 293T cells co-transfected with plasmids encoding STING and NIK. Data are means ± SEM of one experiment run in triplicates out of three independent experiments. **d** Co-immunoprecipitation and immunoblot of HA-STING with Myc-STING in the absence or presence of NIK co-transfected in HEK 293T cells. Results are representative of three independent experiments. **e** Semi-native PAGE immunoblot analysis of STING dimerization in WT and *Nik*^*−/−*^ BMDMs transfected with B-DNA (2 μg/mL) for the indicated time points. Results are representative of two independent experiments. **f** Co-immunoprecipitation and immunoblot of Flag-IRF3 with HA-STING in the absence or presence of Myc-NIK co-transfected in HEK 293T cells. Results are representative of three independent experiments. **g** Immunoblot analysis of STING–IRF3 or STING–TBK1 interactions in WT and *Nik*^*−/−*^ BMDMs transfected with B-DNA (2 μg/mL) for the indicated time points and immunoprecipitated for STING. Results are representative of two independent experiments. *p* < 0.05 is considered significant

To elucidate how NIK was positively regulating the DNA pathway selectively, we hypothesized NIK operated via a signaling component restricted to the DNA pathway. While multiple PRRs have been described to detect DNA species, it is believed that all DNA-dependent signaling to IFN requires the adaptor STING to facilitate downstream signal transmission to the IRF3 transcription factor^4,22^. Co-immunoprecipitation experiments using epitope-tagged NIK, STING, and IRF3 in human embryonic kidney 293T (HEK 293T) cells revealed that NIK interacted with STING as well as IRF3 while confocal analysis showed NIK co-localized with STING (Fig. 3a and Supplementary Figure 3A). NIK also associated with STING at the endogenous level upon B-DNA stimulation (Fig. 3b). Next we wanted to determine the physiological consequence of the NIK–STING interaction. Reporter assays revealed that NIK synergized with STING to increase STING-dependent activation of an IFN-β luciferase reporter (Fig. 3c). Operating differentially in the RNA pathway, however, NIK blocked IFN-β activation mediated by the RNA pathway adaptor, IPS-1 (Supplementary Figure 3B).

In the DNA pathway, STING undergoes dimerization as a prerequisite for IFN activation^23–25^. Ectopic expression of NIK increased the formation of STING–STING complexes and STING dimers (Fig. 3d and Supplementary Figure 3C). In contrast, B-DNA-induced dimerization of STING was impaired in NIK-deficient cells (Fig. 3e). Recent studies have further indicated STING forms high-order aggregates upon activation in the DNA pathway to achieve IFN activation^21,26^. Our data show that NIK could also elevate STING aggregation (Supplementary Figure 3C).

In the DNA pathway, activated STING bridges TBK1 with IRF3 to promote IRF3 phosphorylation-dependent activation^27^. Since NIK also interacted with IRF3 (Fig. 3a), we wanted to determine its physiological relevance. Expression of IRF3 or NIK alone led to minimal activation of the IFN-β luciferase reporter. However, IFN-β activity was synergistically elevated when IRF3 and NIK were co-expressed (Supplementary Figure 3D). Accordingly, the expression of NIK enhanced STING–IRF3 complex formation (Fig. 3f), while STING–IRF3 or STING–TBK1 complex formations were compromised in cells lacking NIK (Fig. 3g).

These findings suggest NIK functions as a positive regulator of the DNA pathway by targeting STING and enhancing its activation resulting in elevated STING signaling complex formations.

### Alternative NF-κB activation heightens DNA pathway signaling

**Fig. 4:**
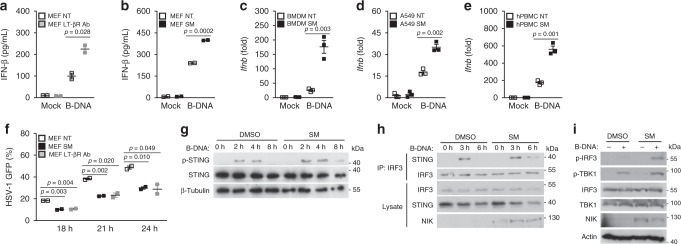
Induction of NIK via alternative NF-κB pathway-activating ligands or pharmacological reagents enhances the DNA pathway. **a**, **b** ELISA for IFN-β in supernatants collected from WT MEFs treated with DMSO (NT), LT-βR agonistic antibody (2 μg/mL) (**a**), or with SMAC mimetic (SM) (1 μg/mL) (**b**) and subsequently transfected with B-DNA (500 ng/mL) for 24 h. Data are means ± SEM of one experiment run in duplicates out of two independent experiments. **c**–**e** Q-PCR of IFN-β mRNA levels in BMDMs (**c**), A549 cells (**d**), or PBMCs (**e**) pretreated with DMSO (NT) or SM (1 μg/mL) for 16 h followed by B-DNA transfection (100 ng/mL) for 4 h. Data are means ± SEM of one experiment run in triplicates out of 2–3 independent experiments. **f** HSV-1 GFP virus populations at the indicated time points in WT MEF cells treated with SM (1 μg/mL), LT-βR agonistic Ab (2 μg/mL), or a DMSO control (NT) for 12 h and subsequently infected with HSV-1 GFP (MOI 0.1). Data are means ± SEM of one experiment run in duplicates out of two independent experiments. **g** Immunoblot analysis of STING phosphorylation (Ser 366) in THP-1 monocytes pretreated with vehicle control (DMSO) or SM (1 μg/mL) for 16 h followed by B-DNA transfection (1 μg/mL) for the indicated time points. Results are representative of two independent experiments. **h** Immunoblot analysis of IRF3-STING interactions in WT MEFs pretreated with DMSO or SM (1 μg/mL) for 16 h and subsequently transfected with B-DNA (2 μg/mL) for the indicated time points. Results are representative of two independent experiments. **i** Immunoblot analysis of IRF3 and TBK1 activation in A549 cells pretreated with vehicle control (DMSO) or SM (1 μg/mL) for 16 h followed by B-DNA transfection (2 μg/mL) for 4 h. Results are representative of three independent experiments. *p* < 0.05 is considered significant

In resting cells, NIK is persistently suppressed by the TRAF3–TRAF2–cIAP1–cIAP2 inhibitory complex as a mechanism to prevent aberrant activation of the alternative NF-κB signaling pathway. Ligation of select TNF receptor super family (TNFRSF) members results in the degradation of TRAF3 and TRAF2, lifting their inhibition of NIK. Likewise, cellular treatment with second mitochondria-derived activator of caspases (SMAC) mimetics (SM) (a small molecule compound which mimics the pro-apoptotic SMAC (aka Diablo) protein to antagonize cIAP function) also results in the increase of NIK expression^15,28,29^. We therefore hypothesized that activation of the alternative NF-κB signaling pathway by either TNFRSF ligation or SM treatment would also enhance IFN activation in the DNA pathway due to their abilities to induce NIK expression. Indeed, MEF cells pre-stimulated with a lymphotoxin beta receptor agonistic antibody (LT-βR Ab) or SM increased NIK expression and B-DNA-mediated induction of IFN compared to untreated cells (Fig. 4a, b and Supplementary Figure 4A). Similarly, SM pretreatment of murine BMDMs, human A549 lung epithelial cells, or primary human peripheral blood monocytes (PBMC) led to enhanced IFN activation in the DNA pathway (Fig. 4c–e and Supplementary Figure 4B). Notably, SM and LT-βR Ab required NIK as they could not elevate the DNA pathway IFN response in cells lacking NIK (Supplementary Figure 4C). Owing to the increase in IFN levels upon LT-βR Ab or SM stimulation, HSV-1-infected MEF cells displayed lower DNA viral burdens when compared to untreated control cells (Fig. 4f).

Upon PRR sensing of cytosolic DNA, STING exits the endoplasmic reticulum and translocates to the Golgi apparatus and ultimately to perinuclear vesicles where it forms punctate structures (Supplementary Figure 4D) and assembles with TBK1^22,30,31^. Imaging flow cytometric analysis indicated that pretreatment of cells with SM further enhanced B-DNA-elicited formation of STING puncta (Supplementary Figure 4E–G). TBK1–STING complex formation results in TBK1 phosphorylation of STING (Ser 366), which leads to the recruitment of IRF3 to STING and allows for TBK1-dependent phosphorylation of IRF3^32^. Indeed, SM pretreatment increased and prolonged the levels of phosphorylated STING (Fig. 4g), resulting in sustained STING-IRF3 recruitment (Fig. 4h) and subsequently elevated IRF3 phosphorylation (Fig. 4i and Supplementary Figure 4H).

In DNA pathway signaling, STING is also subjected to feedback inhibition by various mechanisms including Lysine 48 (K48-linked) ubiquitination, which mediates its proteasomal degradation^33–36^. Interestingly, NIK expression dampened the assembly of K48-linked ubiquitin chains on STING (Supplementary Figure 4I). In support of this observation, SM pretreatment further delayed B-DNA-instigated turnover of STING compared to untreated cells (Supplementary Figure 4J).

Thus, in concert with the DNA pathway, induction of alternative NF-κB signaling/NIK via TNFRSF ligation or pharmacological reagents increases IFN activation by enhancing STING signaling and activation.

### NIK operates independently of IKKα/p100 in the DNA pathway

**Fig. 5:**
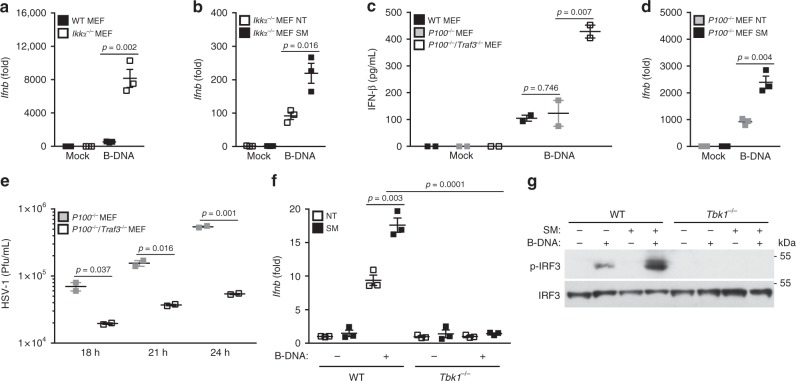
NIK operates independently of the alternative NF-κB signaling components in the DNA pathway. **a**, **b** Q-PCR of IFN-β mRNA levels in WT and *Ikkα*^*−/−*^ MEFs (**a**) or in *Ikkα*^*−/−*^ MEFs pretreated with DMSO (NT) or SM (1 μg/mL) for 16 h (**b**) and subsequently transfected with B-DNA (500 ng/mL) for 4 h. Data are means ± SEM of one experiment run in triplicates out of 2–3 independent experiments. **c** IFN-β ELISA from supernatants of WT, *P100*^*−/−*^, and *P100*^*−/−*^*/Traf3*^*−/−*^ MEFs collected 24 h after B-DNA transfection (500 ng/mL). Data are means ± SEM of one experiment run in duplicates out of two independent experiments. **d** Q-PCR of IFN-β mRNA in *P100*^*−/−*^ MEFs pretreated with DMSO (NT) or SM (1 μg/mL) for 16 h followed by B-DNA transfection for 4 h. Data are means ± SEM of one experiment run in triplicates out of two independent experiments. **e** Viral titers determined via plaque assay from supernatants of *P100*^*−/−*^ and *P100*^*−/−*^*/Traf3*^*−/−*^ MEFs infected with HSV-1 (MOI 0.1) at the indicated time points. Data are means ± SEM of one experiment run in duplicates out of two independent experiments. **f** Q-PCR of IFN-β mRNA induction in WT and *Tbk1*^*−/−*^ MEFs pretreated with DMSO (NT) or SM (1 μg/mL) for 16 h and subsequently transfected with B-DNA (500 ng/mL) for 4 h. Data are means ± SEM of one experiment run in triplicates out of three independent experiments. **g** Immunoblot analysis of IRF3 activation in WT and *Tbk1*^*−/−*^ MEFs pretreated with DMSO (NT) or SM (1 μg/mL) for 16 h and subsequently transfected with B-DNA (2 μg/mL) for 2 h. Results are representative of two independent experiments. *p* < 0.05 is considered significant

Our data indicate that NIK positively regulates IFN in the DNA pathway, which can also be synergized via crosstalk with the alternative NF-κB pathway. As a critical component of the alternative NF-κB pathway, NIK signals via inhibitor of kappa B kinase alpha (IKKα, aka Chuk) to phosphorylate the p100 NF-κB subunit (aka NFkB2), which leads to its activation^14,37–40^. We therefore hypothesized that, in the DNA pathway, NIK would utilize both its signaling partner, IKKα, and the downstream substrate, p100, to achieve IFN activation as these downstream components are also activated in response to B-DNA stimulation (Supplementary Figure 5A). Surprisingly, IKKα did not mirror NIK in that IKKα-deficient MEFs displayed an increase in IFN activation in response to B-DNA (Fig. 5a and Supplementary Figure 5B). Correspondingly and in contrast to NIK-deficient MEFs, MEFs lacking IKKα harbored lower DNA viral burdens after infection (Supplementary Figure 5C–D). To further delineate whether NIK required IKKα in the DNA pathway, IKKα-deficient MEFs were treated with SM followed by B-DNA transfection. SM (NIK induced) mediated activation of IFN was still achieved even in the absence of IKKα, suggesting that IKKα is dispensable in the DNA pathway (Fig. 5b). Similarly, STING-dependent activation of IFN was not suppressed in IKKα-deficient MEFs nor did a NIK mutant that does not interact with IKKα (NIK Aly) show impaired synergy with STING in activating IFN (Supplementary Figure 5E–F).

The alternative NF-κB transcription factor component, p100, was also dispensable in eliciting induction of IFN in the DNA pathway (Supplementary Figure 5B and 5G). However, when NIK was induced, in doubly deficient *Traf3*^−/−^*P100*^*−/−*^ MEFs or in *P100*^*−/−*^ MEFs pretreated with SM, elevated IFN levels were restored, indicating that NIK-dependent activation of IFN did not require p100 (Fig. 5c, d and Supplementary Figure 5H). Elevated IFN levels displayed in the *Traf3*^−/−^*P100*^*−/−*^ MEFs consequently provided greater protection against DNA viral infection compared to *P100*^*−/−*^ MEFs (Fig. 5e).

Our results reveal that NIK activates IFN in a manner independent of its signaling partner, IKKα, and independent of the alternative NF-κB transcription co-factor, p100. Since NIK could also interact with IRF3 and increase its activation (Fig. 3a and Supplementary Figure 3D), we reasoned that NIK was simply a direct kinase for IRF3. Induction of NIK via SM pretreatment led to increased IFN activation and IRF3 phosphorylation in the DNA pathway. However, cells lacking the upstream IRF3 kinase, TBK1, showed no elevation in IFN activation or IRF3 phosphorylation when pretreated with SM (Fig. 5f, g and Supplementary Figure 5I). Thus our findings indicate that NIK is not a kinase for IRF3 and activates IFN in the DNA pathway via a mechanism that is separate from its role in the alternative NF-κB pathway.

### NIK requires its kinase function and auto-phosphorylation

**Fig. 6:**
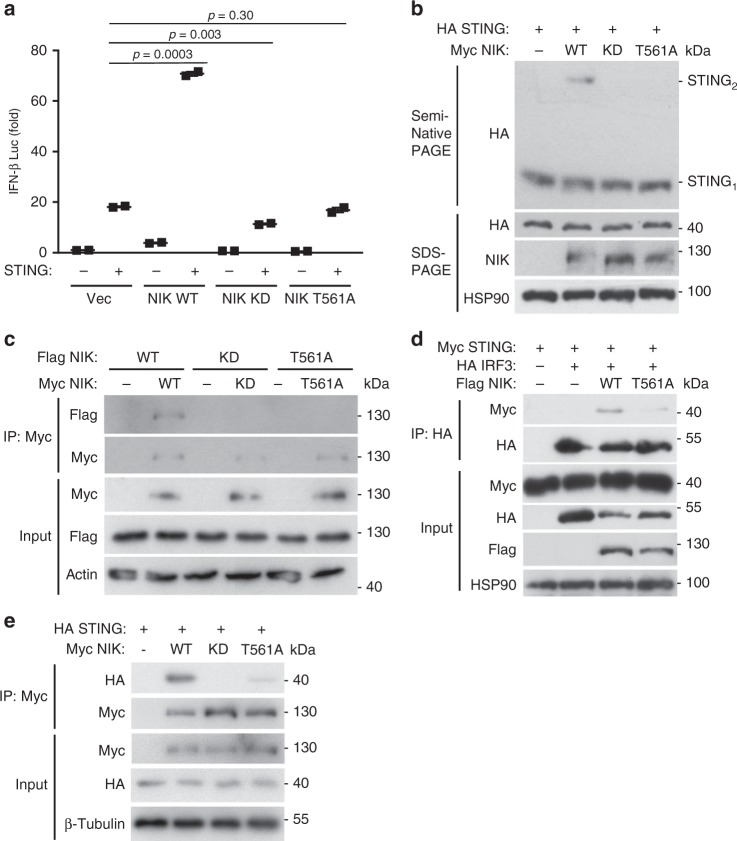
NIK requires its kinase function and phosphorylation at Thr 561 to potentiate STING activation in the DNA pathway. **a** IFN-β luciferase reporter assay in HEK 293T cells co-transfected with plasmids encoding STING and wild-type NIK, (NIK WT), kinase dead NIK (NIK KD), or a Thr 561 phospho-acceptor mutant NIK (NIK T561A). Data are means ± SEM of one experiment run in duplicates out of three independent experiments. **b** Semi-native PAGE immunoblot analysis of STING dimerization in HEK 293T cells co-transfected with HA-STING and Myc-WT, KD, or T561A NIK plasmids. Results are representative of two independent experiments. **c** Co-immunoprecipitation and immunoblot between Myc-WT, KD, or T561A NIK and Flag-WT, KD, or T561A NIK co-transfected in HEK 293T cells. Results are representative of three independent experiments. **d** Co-immunoprecipitation and immunoblot of HA-IRF3 with Myc-STING in the absence or presence of Flag-WT or T561A NIK co-transfected in HEK 293T cells. Results are representative of two independent experiments. **e** Co-immunoprecipitation and immunoblot of Myc-WT, KD, or T561A NIK with HA-STING co-transfected in HEK 293T cells. Results are representative of three independent experiments. *p* < 0.05 is considered significant

To date, IKKα has been demonstrated to be the only known substrate for the kinase NIK. However, our data indicates that NIK did not require IKKα to activate IFN in the DNA pathway. We therefore hypothesized NIK was operating in a kinase-independent manner to facilitate STING-dependent signaling. However, IFN-β luciferase reporter assays utilizing a WT and a kinase-dead mutant of NIK (KK429/430AA) (NIK KD) showed that, while WT NIK synergized with STING to enhance IFN activation, the NIK KD mutant failed to support STING-mediated activation of IFN (Fig. 6a). Similar findings were observed for IRF3 (Supplementary Figure 6A). In line with these findings, NIK required its kinase function to increase STING dimerization or aggregation (Fig. 6b and Supplementary Figure 6B).

Our data indicate that while NIK activates STING in a manner independent of its canonical substrates, IKKα/p100, it still requires its kinase function. However, ectopic co-expression of NIK with STING, TBK1, or IRF3 did not reveal any obvious shift in their molecular weights when examined via immunoblotting, suggesting that those signaling components were not direct substrates of NIK (Fig. 3d, f). To gain a better understanding for the necessity of the kinase function of NIK in innate antiviral signaling pathways, we noted an emerging theme of high-order protein assembly or oligomerization as a requisite in instigating IFN activation. For example, RIG-I/MDA5 as well as cGAS PRRs have been demonstrated to form ligand-induced tetramers and dimers, respectively^41–44^. The IPS-1 and STING adaptors facilitate downstream signal transmission by forming aggregates and/or dimers^24,26,45,46^. TBK1 and IRF3 both undergo phosphorylation-dependent dimerization in order to induce IFN^47–51^. In line with this signaling paradigm, we hypothesized that the substrate for NIK was itself and that NIK required its kinase function to confer auto-phosphorylation, which would lead to its subsequent stable dimerization as a mechanism to achieve STING activation. Indeed, NIK required its kinase function to facilitate NIK dimerization as a NIK KD mutant failed to self-associate or oligomerize (Fig. 6c and Supplementary Figure 6C). NIK had been previously reported to undergo auto-phosphorylation (Thr 561 in mouse, Thr 559 in human) in order to activate IKKα in the alternative NF-κB pathway^52^. To determine whether NIK also underwent auto-phosphorylation in the DNA pathway, we stimulated human A549 lung epithelial cells with B-DNA in a timed course experiment. Immunoblotting analysis indicated that NIK was indeed subjected to Thr 559 phosphorylation in response to B-DNA (Supplementary Figure 6D). In agreement with our hypothesis that NIK kinase function is important for auto-phosphorylation-induced dimerization, a NIK Thr 561 phospho-acceptor mutant also displayed an impaired capacity to dimerize (Fig. 6c). Consequently, only WT NIK (dimer competent) could increase STING- and IRF3-dependent activation of IFN, whereas the NIK T561A mutant (dimer incompetent) could not (Fig. 6a and Supplementary Figure 6A). However, when the NIK T561A mutant (which has intact kinase function) was co-expressed with the NIK KD mutant (which is competent in undergoing Thr 561 phosphorylation), STING-mediated activation of IFN was remediated, further suggesting that the ability of NIK to phosphorylate and accept phosphorylation is essential in enhancing STING activation (Supplementary Figure 6E). The crucial role of phosphorylation-dependent dimerization of NIK was further demonstrated as it was revealed that the NIK T561A mutant could not promote the formation of STING dimers nor enhance the formation of STING–IRF3 complexes (Fig. 6b, d). Mechanistically, the oligomeric conformation of NIK favored its ability to activate IFN via STING as both the KD and phospho-acceptor mutant of NIK were defective in interacting with STING (Fig. 6e). Together, these results uncover a critical role for NIK kinase function and self-phosphorylation, underscoring the essential role of protein oligomerization in driving signal transduction events.

### NIK plays an essential role in restricting DNA virus infection in vivo

**Fig. 7:**
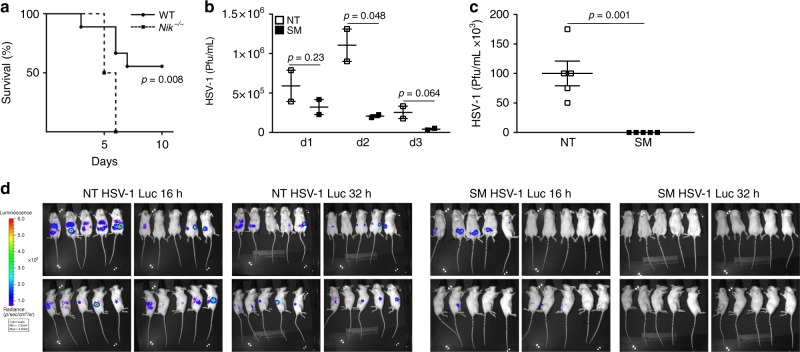
NIK expression in vivo supports enhanced host defense against DNA virus infections. **a** Survival curve in WT (*n* = 9) and *Nik*^*−/−*^ (*n* = 8) mice retro-orbitally infected with HSV-1 (1 × 10^9^ Pfu/mouse). **b** Plaque assays measuring viral titers in WT mice treated with vehicle control DMSO (NT) or SM (*n* = 4 per group) followed by HSV-1 infection (2.7 × 10^7^ Pfu/mouse) for 4 h taken from vaginal lavages at the indicated time points. Data are means ± SEM run in duplicates. **c** Plaque assays determining viral titers in the spleens of WT mice treated with vehicle control DMSO (NT) or SM (*n* = 5 per group) and subsequently infected with HSV-1 (2 × 10^6^ Pfu/mouse) 24 h after infection. The number of plaques from each control-treated or SM-treated spleen are shown. **d** Bioluminescent in vivo imaging of WT mice treated with vehicle control DMSO (NT) or SM (*n* = 5 per group) followed by infection with HSV-1 encoding luciferase (500 Pfu/mouse) for the indicated time points. *p* < 0.05 is considered significant

Our data show that, in cells lacking NIK, IFN induction in response to DNA viral infections or B-DNA stimulation is impaired, resulting in the failure to control DNA virus replication. To determine the physiological relevance of NIK in response to DNA virus infection, WT and NIK-deficient mice were infected with HSV-1 and monitored for loss in body weight. While most of the WT mice survived the HSV-1 challenge, all of the mice lacking NIK displayed a heightened sensitivity to HSV-1, presenting with lower survival rates and increased weight loss (Fig. 7a and Supplementary Figure 7A–B). As these findings highlight the critical role of NIK in conferring host defense against DNA viral infections in vivo, we reasoned that elevating expression of NIK via pharmacological application with SMAC mimetics could also inhibit DNA virus replication in vivo. In an HSV-1 vaginal infection model, female mice administered with SM followed by intravaginal challenge with HSV-1 harbored reduced viral titers in comparison to untreated infected mice (Fig. 7b). Likewise, administration of SM resulted in lowered viral titers of HSV-1 in the spleens of SM-treated mice compared to those of untreated mice (Fig. 7c). Lastly, utilizing in vivo bioluminescence imaging, we found that administration of SM further enhanced the clearance of HSV-1 compared to matched animals that were not treated (Fig. 7d). Together these data indicate a critical in vivo role for NIK and support a therapeutic potential for SMs in controlling DNA virus infections.

## Discussion

Although viral infections trigger the innate immune induction of IFN, it is becoming increasingly clear that the mode of IFN activation is dependent upon the PRR type that is engaged. Indeed, cytosolic detection of RNA virus genomes or RNA species by the RIG-I like helicases activates IFN via the IPS-1 adaptor (RNA pathway), while cytosolic sensing of DNA virus genomes or DNA species by the DDX41 helicase, cGAS, or IFI16 activates IFN utilizing the STING adaptor (DNA pathway). However, additional mechanisms that define how these pathways are separately or differentially modulated are largely uncharacterized. Previous studies demonstrated that signaling in the RNA pathway required TRAF3 to facilitate IFN activation. Our current work shows that TRAF3 surprisingly plays a direct opposite role in the DNA pathway where it functions to inhibit IFN activation.

TRAF3 exists as part of a multiprotein complex containing TRAF2, cIAP1, and cIAP2 that function in restricting the activation of the alternative NF-κB pathway^11,12^. We found that, like TRAF3, TRAF2 also played an important role in positively regulating the RNA pathway and suppressing the DNA pathway. While the cIAP1 and cIAP2 components have been previously shown to mediate IFN activation in the RNA pathway^13^, our results indicating that they inhibit the DNA pathway is consistent with the phenotype observed with their interacting partners, TRAF3 and TRAF2. Therefore, IFN activation in the RNA vs. the DNA pathway is regulated both positively and negatively, respectively, by the modulators of the alternative NF-κB signaling pathway. In this alternative NF-κB pathway, TRAF3, TRAF2, cIAP1, and cIAP2 cooperate to promote the persistent degradation of the alternative NF-κB inducing kinase, NIK. A recent study reported that NIK serves as a negative regulator of IFN in the RNA pathway^19^. These findings align with our observation that cells lacking the NIK inhibitors, TRAF3, TRAF2, cIAP1, or cIAP2, fail to induce a robust IFN response in the RNA pathway. Thus TRAF3/TRAF2/cIAP1/cIAP2 may function via a two-pronged mechanism in the RNA pathway where they operate as (1) adaptor molecules to mediate downstream signal transmission to IFN and as (2) inhibitors of the inhibitor, NIK^28^. Accordingly, work by other groups examining feedback inhibition have shown that TRAF3 is subjected to degradation to terminate RNA pathway signaling, which coincides with NIK accumulation^53^. Our findings uncover a similar phenomenon in the DNA pathway where the abundance of TRAF3 is reduced during signaling with the concomitant accumulation of NIK. This also suggests a two-pronged mechanism of regulation in the DNA pathway where the suppressive role of TRAF3 as a steady state inhibitor is lifted, allowing for the activating role of NIK to come into play.

Given the relatively slow kinetics of TRAF3 turnover and NIK accumulation in the DNA pathway, it may be plausible that NIK functions to sustain IFN activation while playing a less prominent role in immediate or early DNA PRR signaling. Indeed, the role of NIK in inducing IFN or in maintaining STING complexes were markedly noticeable at later time points. Furthermore, our data showcased a delayed interaction between NIK and the DNA signaling adaptor, STING. However, it should be noted that there are technical limitations in detecting accumulated NIK (via immunoblotting using current commercially available antibodies) whose expression levels are typically low in different cell types even upon activation of alternative NF-κB or cytosolic DNA-sensing pathways. To this end, the deficiency in IFN activation or the inability of STING to optimally dimerize upon B-DNA stimulation in cells lacking NIK was still apparent at earlier time points even though NIK protein was not easily detected. Thus a second, more likely possibility is that NIK may play a more prominent role in early STING activation, driving later-stage STING signaling.

During end-stage signaling in the DNA pathway, STING has also been reported to undergo feedback inhibition via K48-linked ubiquitination-mediated proteasomal degradation as a regulatory mechanism to terminate excessive IFN activation^33–35^. As our data indicate that NIK expression suppresses the delivery of K48-linked ubiquitin chains onto STING and enhances STING stability, it is possible that NIK may further play a role in restricting the interaction between potential E3 ubiquitin ligases that target STING for degradation. Alternatively, NIK could be recruiting de-ubiquitinating enzymes that de-conjugate K48-linked ubiquitin chains from STING^36,54^. It has been shown that inactive rhomboid protein 2 (iRhom2) also enhances DNA pathway activation via a two-pronged mechanism by facilitating STING trafficking from the endoplasmic reticulum to perinuclear vesicles and by recruiting the K48-linked de-ubiquitinating enzyme, eukaryotic translation initiation factor 3 subunit F (EIF3F, aka EIF3S5), to enhance STING stability^36^. However, iRhom2 function is not restricted to the DNA pathway as it was recently demonstrated that it also enhanced IPS-1 stability to increase RNA pathway signaling^55^. Although iRhom2 functions as a positive regulator of both the DNA and RNA pathways, NIK selectively functions to positively regulate the DNA pathway while serving as a negative regulator of the RNA pathway. Nevertheless, further studies will be necessary to determine whether NIK cooperates with iRhom2/EIF3F to modulate STING function in the DNA pathway.

The critical role of NIK was further exemplified by the capacity of the alternative NF-κB pathway to crosstalk with the DNA pathway. Interestingly, while NIK-mediated inhibition of the RNA pathway was shown to require alternative NF-κB pathway signaling components^19^, our data indicated NIK activates the DNA pathway in an alternative NF-κB pathway-independent manner. We found that neither the classical NIK substrate and signaling partner, IKKα, nor its downstream alternative NF-κB transcription co-factor precursor, p100, were  essential in supporting IFN activation in the DNA pathway. Unexpectedly, and in contrast to NIK-deficient cells, cells lacking IKKα displayed even greater levels of IFN and consequently harbored lower DNA virus loads. In alternative NF-κB signaling, NIK phosphorylates IKKα that leads to the activation of its kinase function and confers its ability to phosphorylate p100 to ultimately yield the formation of an unrestrained, competent transcription factor complex. In a negative feedback loop, the activated IKKα also phosphorylates NIK at a C-terminal cluster consisting of Ser 809, 812, and 815, promoting its degradation in a TRAF3/TRAF2/cIAP1/cIAP2-independent manner. As such, cells lacking IKKα fail to degrade accumulated NIK^56,57^. Thus both the steady-state inhibitors as well as the feedback inhibitor of NIK inhibit IFN activation in the DNA pathway. Importantly, our findings reveal a new, previously undescribed pathway for NIK that is separate from its role in the alternative NF-κB pathway, in facilitating IFN activation in innate antiviral immune signaling.

In addition to NIK enhancing STING activation, our data showed that ectopic expression of NIK with IRF3 also resulted in the synergistic activation of IFN. Although NIK interacted with IRF3, our results revealed that NIK was not a kinase for IRF3 and confirmed that TBK1 was essential in phosphorylating IRF3 in the DNA pathway^58,59^. Nevertheless, since IRF3 is a shared component in both the DNA/STING and RNA/IPS-1 pathways, we found it curious as to how NIK supported IRF3 activation as opposed to inhibition when co-expressed with IRF3. In the RNA pathway, the IPS-1 adaptor functions to connect the upstream RIG-I or MDA5 PRRs with TRAF3^10^. This is different in the DNA pathway where it is believed that the STING adaptor operates further downstream, bridging the interaction between TBK1 and IRF3^27^. Alternatively, new studies further indicate that phosphorylated IPS-1 can also recruit IRF3^32^. Additional experiments examining how NIK affects IPS-1 at the posttranslational level may reveal how NIK negatively regulates the RNA/IPS-1 pathway. Given that NIK plays a role in maintaining or enhancing STING complex formations including those complexes containing STING/TBK1/IRF3, it is possible that NIK overexpression potentiated the basal activation of endogenous STING, facilitating the recruitment of TBK1 with the ectopically expressed IRF3 to drive IFN activation. NIK is also known to interact with TBK1, also a shared component in both the RNA and DNA signaling pathways. Interestingly, it was recently reported that TBK1 played a key regulatory role in the alternative NF-κB pathway where it phosphorylated NIK at Ser 862 causing its degradation and the termination NF-κB signaling^60^. Although reminiscent of the modulatory role of IKKα in the alternative NF-κB pathway^57^, the potential TBK1-NIK regulatory mechanisms in the DNA pathway will require further investigation.

A common theme in multiple signal transduction pathways is of protein phosphorylation and oligomerization. Indeed, the RNA sensors RIG-I and MDA5 have been demonstrated to form tetramers while the cGAS or IFI16 DNA sensors form dimers or filamentous-like aggregates upon ligand binding. The IPS-1 and STING adaptors are further subjected to phosphorylation-dependent aggregation and/or dimerization to facilitate downstream signal transmission. Similarly, both the TBK1 kinase and IRF3 transcription factor undergo dimerization in a phosphorylation-dependent manner to activate IFN^61,62^. Although the downstream substrate of NIK, IKKα, was not required in the DNA pathway, the kinase function of NIK was nevertheless revealed to be critical in activating the IFN response. We did not find STING, TBK1, nor IRF3 to be the obvious substrates for NIK; however, our data suggested that the kinase activity of NIK was essential in supporting its ability to self-associate or oligomerize. Consequently, a KD mutant of NIK failed to interact with STING and thus could not promote its activation. Several kinases including TBK1 have been reported to undergo auto- or trans-phosphorylation in order to achieve a stable dimeric conformation that confers optimal signaling capabilities^49^. Seminal studies with NIK uncovered that key residues in its kinase domain were important in positively regulating its ability to activate the alternative NF-κB pathway. Indeed, Thr 561 (Thr 559 in human), located within the activation loop of NIK’s kinase domain, was demonstrated to be an important phospho-acceptor site and Ala substitution (T561A) failed to induce IKKα activity and subsequent NF-κB activation^52^. In support of a phosphorylation-mediated oligomerization theme in innate signaling, the NIK phospho-acceptor mutant, behaving similarly to the KD NIK mutant, was compromised in forming competent dimers. As such, the interaction between NIK KD or NIK T561A and STING was much weaker compared to NIK WT, explaining its impaired ability to activate STING or facilitate STING complex formations. These mechanistic findings highlight and further support the essential role of protein oligomerization as a mechanism to drive signal transduction pathways.

In the clinic, IFN therapies are widely used to treat multiple viral infections^63,64^. Interestingly, the alternative NF-κB pathway plays an important synergistic role in crosstalking with the DNA pathway. Indeed, previous works found a link between the alternative NF-κB pathway and DNA virus-induced activation of IFN^65^. Our results revealed and defined the specific role of the alternative NF-κB inducing kinase,  NIK in the DNA pathway and further elucidated the mechanism by which it facilitates host defense against DNA virus infections. The elevation of NIK via ligands that activate TNF superfamily of receptors or by administration of SMAC mimetics have therapeutic potential in elevating the host IFN response as animals that were pretreated with SMAC mimetics followed by HSV-1 challenge presented with lower viral loads compared to non-treated infected animals. Future studies will be necessary to determine the therapeutic applications of SMAC mimetics in combating DNA virus infections.

In summary, we have identified a unique phenomenon where the same molecule(s) functions to oppositely regulate the host IFN pathway in a manner that depends on the nucleic acid type detected. We also uncover a key component of the alternative NF-κB pathway that operates in a separate, alternative NF-κB-independent fashion to activate IFN in the DNA pathway. Our findings further speak of the importance of protein oligomerization in potentiating signal transduction events in innate antiviral immunity. Lastly, our work lends to a conceptual framework in developing potential therapeutic applications in targeting certain viral infections.

## Methods

### Mice and cell culture

Generation of *Traf3*^*−/−*^, *Nfkb2*^*−/−*^, *Traf3*^*−/−*^*Nfkb2*^*−/−*^, and *Map3K14*^*−/−*^ mice were previously described^12,66,67^. All mice were maintained and bred in specific pathogen-free conditions at the University of California, Los Angeles, and experiments were performed within the parameters of the protocol approved by the Animal Research Committee. *Traf2*^*−/−*^ MEFs were a kind gift from Wen-Chen Yeh (University of Toronto). *Sting*^*−/−*^ MEFs stably reconstituted with STING-GFP were kindly provided by Nan Yan (University of Texas Southwestern Medical Center). *Chuk*^*−/−*^, *Tbk1*^*−/−*^, and *Birc2*^*−/−*^*Birc3*^*−/−*^ MEFs were previously reported^12,57,59^. HEK 293T cells, A549 cells, and THP-1 monoctyes were from American Type Culture Collection (ATCC). BMDMs were harvested from the femurs and tibias of 6–8-week-old mice and differentiated for 7 days in Dulbecco’s modified Eagle’s medium (DMEM; Mediatech Inc.) containing 10% fetal bovine serum (FBS) and macrophages colony-stimulating factor. HEK 293T cells, A549 cells, and MEFs were cultured in DMEM supplemented with 5% FBS and 1% penicillin/streptomycin. THP-1 monocytes were cultured in RPMI-1640 (Mediatech Inc.) supplemented with 10% FBS and 0.05 mM 2-mercaptoethanol.

### Reagents

Poly (I:C), poly (dA:dT), ISD, and cGAMP were purchased from Invivogen. Calf thymus DNA was purchased from Sigma. Vaccinia virus DNA was purchased from ATCC. Cell transfections were performed using Lipofectamine 2000 while siRNA targeting NIK (Bioneer) was transfected using Lipofectamine RNAi Max (Invitrogen). SMAC mimetic (NVP-LCL161_NX-g) was generously provided by Xiaodong Wang (University of Texas Southwestern Medical Center). IFN-α4 and IFN-β enzyme-linked immunosorbent assay (ELISA) kits were obtained from PBL Biomedical Laboratories; ELISAs were performed according to the manufacturer’s recommended protocols. Lymphotoxin beta receptor agonist antibody was purchased from Alexis Biochemicals. Antibodies were obtained from the following companies: STING, IRF3, NIK, TBK1, TRAF2, cIAP1, IKKα, p100, cGAS, β-Actin, phospho-STING (S366), phospho-IRF3 (S396), phospho-TBK1 (S172), phospho-IKKα (S176), and phospho-STAT1 (Y701) (Cell Signaling); Flag, β-tubulin (Sigma Aldrich); STAT1, HSV-1 ICP4, HSP90, TRAF3, cIAP2, phospho-NIK (T559), Myc, HA (Santa Cruz Biotechnology); HSV-1 glycoprotein D (Abcam); and ZDHHC1 (ThermoFisher). Plasmids encoding TRAF3, NIK, IRF3, STING, IPS-1, TBK1, and K48 only ubiquitin have been described elsewhere^10,12,17,57^. NIK Aly, KD, and T561A mutants were generated using the Quikchange Site-Directed Mutagenesis Kit (Agilent).

### Immunoassays

For immunoblot analysis, cells were harvested in ice-cold NP-40 lysis buffer (50 mM Tris-Cl pH 7.4, 150 mM NaCl, 1 mM EDTA, 1% NP-40) supplemented with complete protease inhibitors (Roche). For immunoprecipitation experiments, precleared lysates from transfected or stimulated cells were incubated overnight with appropriate antibodies at 4 °C followed by the addition of protein A agarose beads (Roche) for 4 h at 4 °C. Captured protein complexes were washed three times with NP-40 lysis buffer containing 250 mM NaCl and then eluted with 2× Laemmli sample buffer (Bio-Rad) containing β-mercaptoethanol. Samples were boiled at 95 °C for 5 min followed by sodium dodecyl sulfate-polyacrylamide gel electrophoresis (SDS PAGE) and immunoblotting. For semi-native SDS PAGE, whole-cell lysates were mixed with 2× native sample buffer (65.8 mM Tris-Cl pH 6.8, 40% glycerol, 0.01% bromophenol blue) without β-mercaptoethanol and were run on SDS gels without boiling. For semi-denaturating detergent agarose gel electrophoresis, lysates were separated on 1.5% agarose gels followed by immunoblotting as described. For detecting STING ubiquitination, immunoprecipitates were subjected to additional washes containing 1 M urea as previously described^68^. Proteins were detected via enhanced chemiluminescence (Pierce). Uncropped immunoblotting data are included in Supplementary Figure 8.

### Virus infections and assays

VSV (Indiana Serotype) expressing green fluorescent protein (GFP) was a kind gift from Glen Barber (University of Miami School of Medicine). HSV-1 and KOS strain expressing GFP in frame with the ICP0 protein between amino acids 104 and 105 was generously provided by William Halford (Southern Illinois University School of Medicine). HSV-1, F strain expressing GFP, and firefly luciferase was kindly provided by Chunfu Zheng (Wuhan Institute of Virology, Chinese Academy of Sciences). Creation of an MHV-68 virus expressing luciferase under an M3 promoter (M3FL) was previously described. SeV (Cantell strain, VR-907) was purchased from ATCC. Viral titers were determined using standard plaque assays with Vero cells. Virally infected GFP-positive cells were determined using standard flow cytometric analysis while virally infected luciferase harboring cells were detected via a luminometer. For survival assays, sex- and age-matched WT and NIK-deficient mice were anesthetized by isoflurane and infected with 1 × 10^9^ plaque-forming units (Pfu) HSV-1 via retro-orbital injection. Animal weight was monitored daily and mice that lost >20% of their initial weight or those that showed signs of morbidity were euthanized according to UCLA approved protocols. For the HSV-1 intra-vaginal model of infection in WT C57/B6 mice, female mice received a 1 mg subcutaneous injection of Medroxyprogesterone (Sicor), 48 h prior to infection. Twenty four hours prior to infection, mice were anesthetized using an intraperitoneal (IP) injection of ketamin/xylazine and were administered 0.016 mg of SMAC mimetic in 5% dimethyl sulfoxide intravaginally using a 20 μL pipet. On day 0, mice were re-treated with SMAC mimetic and 4 h later infected with 2.9 × 10^7^ Pfu/mouse of HSV-1 strain KOS (ATCC). Twenty four hours later, mice were anesthetized with isoflurane on day 1 to measure vaginal virus titers by vaginal cavity lavage and subsequently re-treated with SMAC mimetic. On days 2 and 3, mice were similarly anesthetized with isoflurane prior to collection of vaginal lavage samples. For in vivo bioluminescent infection and imaging, WT C57/B6 mice were first anaesthetized by IP with 200 mg/kg ketamine, 4 mg/kg xylazine in phosphate-buffered saline (PBS). HSV-1 GFP Luc (500 pfu) in 200 uL of PBS was administered by IP. On day 3 following infection, mice were imaged using the in vivo imaging system (IVIS, Xenogen). Briefly, mice were anesthetized with isoflurane and administered 3 mg D-luciferin/mouse by IP prior to imaging. Grayscale photographs and color images of imaged mice were superimposed with LivingImage (Xenogen) and Igor (Wavemetrics) programs. The mice were imaged on dorsal, ventral, right, and left side until the maximal luminescence passed. The peak intensity of average and maximum photon flux value was measured for each mouse at every angle and expressed as photons/sec/cm^2^/steradian. These values were averaged for each group of mice.

### Quantitative PCR (Q-PCR)

RNA was isolated using TRIzol reagent (Invitrogen) and converted to cDNA using iScript (Bio-Rad). Q-PCR analysis was performed using the iCycler thermocycler. Transcript abundance was first normalized to that of mRNA encoding the ribosomal protein, L32 for murine cells (MEFs or BMDMs), or the ribosomal protein, 36B4 for human cells (A549 and PBMC) and then normalized against values for unstimulated or uninfected controls. Primer sequences are as follows: murine IFNb Fwd AGCTCCAAGAAAGGACGAACAT, Rev GCCCTGTAGGTGAGGTTGATCT; human IFNb Fwd TGTGGCAATT_GAATGGGAGGCTTGA, Rev CGGCGTCCTCCTTCTGGAACTG; L32 Fwd AAGCGAAACTGGCGGAAAC, Rev TAACCGATGTTGGGCATCAG; and 36B4 Fwd TCGAACACCTGCTGGATGAC, Rev CCACGCTGCTGAACATGCT.

### Luciferase assays

Cells were transfected with the specified constructs, along with a Firefly luciferase pGL2-basic reporter plasmid and Renilla luciferase for normalization. Cells were lysed on ice in 1× passive lysis buffer and luciferase values were quantified on a luminometer using a Dual Luciferase Assay System Kit (Promega).

### Immunofluorescence

HEK 293T cells were transfected with HA-STING and Myc-NIK expression vectors. After overnight culture, cells were fixed with 4% paraformaldehyde. Cells were permeabilized with 0.3% Triton-X 100 in PBS and non-specific antibody binding was blocked with 10% Goat and Donkey serum. Cells were then stained with Mouse anti-Myc antibody (Santa Cruz Biotechnology SC-40) and Rabbit anti-HA antibody (Santa Cruz Biotechnology SC-805) diluted 1/50 in 0.1% Triton-X 100, 1% bovine serum albumin (BSA), and in PBS at 4 °C overnight. Cells were then stained with Goat anti-mouse Dylight-649 (Biolegend 405312) and Donkey anti-rabbit Dylight-488 (Biolegend 406404) diluted 1/100 in 0.1% Triton-X 100, 1% BSA, and in PBS at room temperature for 1 h. Slides were mounted with ProLong Gold antifade with 4,6-diamidino-2-phenylindole (ThermoFisher Scientific). Staining specificity was confirmed by the absence of signal in empty vector-transfected cells. Images were acquired on a Leica TCS SP5 II AOBS confocal microscope at the UCLA MIMG microscopy core facility. Images were analyzed using the Fiji distribution of the ImageJ software^69^.

### Imaging flow cytometry

*Sting*^*−/−*^ MEFs stably expressing STING-GFP were stimulated for 0, 30, 60, or 120 min with 500 ng/mL B-DNA. Cells were then trypsinized and fixed for 15 min at room temperature in 2% paraformaldehyde. Cells were acquired on an ImagestreamX Mark II imaging flow cytometer at the UCLA Jonsson Comprehensive Cancer Center and Center for AIDS Research Flow Cytometry Core Facility. The acquired events were analyzed using the IDEAS software (EMD Millipore). Cells were separated based on the puncta detection in the STING-GFP channel using the spot wizard function. Events with a high number of puncta were determined to be activated cells based on previous immunofluorescence staining.

### Statistical analysis

Statistical significance for ELISA, Q-PCR, luciferase reporter assay, plaque assay, flow cytometry (for detecting viral load), and imaging flow cytometry (for detecting STING translocation/puncta formation) was determined via Student’s *t* test. Statistical significance in examining survival among WT and *Nik*^*−/−*^ mice was performed via the Log-rank (Mantel–Cox) test. A *p* value of <0.05 is considered statistically significant.

### Data availability

The data that support the findings of this study are available from the corresponding author upon request.

## Electronic supplementary material

Supplementary Information

## References

[1] Iwasaki A, Medzhitov R (2015). Control of adaptive immunity by the innate immune system. Nat. Immunol..

[2] Pandey S, Kawai T, Akira S (2014). Microbial sensing by Toll-like receptors and intracellular nucleic acid sensors. Cold Spring Harb. Perspect. Biol..

[3] Akira S, Uematsu S, Takeuchi O (2006). Pathogen recognition and innate immunity. Cell.

[4] Barber GN (2014). STING-dependent cytosolic DNA sensing pathways. Trends Immunol..

[5] Broz P, Monack DM (2013). Newly described pattern recognition receptors team up against intracellular pathogens. Nat. Rev. Immunol..

[6] Paludan SR, Bowie AG (2013). Immune sensing of DNA. Immunity.

[7] Häcker H (2006). Specificity in Toll-like receptor signalling through distinct effector functions of TRAF3 and TRAF6. Nature.

[8] Oganesyan G (2006). Critical role of TRAF3 in the Toll-like receptor-dependent and -independent antiviral response. Nature.

[9] Paz S (2011). A functional C-terminal TRAF3-binding site in MAVS participates in positive and negative regulation of the IFN antiviral response. Cell Res..

[10] Saha SK (2006). Regulation of antiviral responses by a direct and specific interaction between TRAF3 and Cardif. EMBO J..

[11] Vallabhapurapu S (2008). Nonredundant and complementary functions of TRAF2 and TRAF3 in a ubiquitination cascade that activates NIK-dependent alternative NF-kappaB signaling. Nat. Immunol..

[12] Zarnegar BJ (2008). Noncanonical NF-kappaB activation requires coordinated assembly of a regulatory complex of the adaptors cIAP1, cIAP2, TRAF2 and TRAF3 and the kinase NIK. Nat. Immunol..

[13] Mao AP (2010). Virus-triggered ubiquitination of TRAF3/6 by cIAP1/2 is essential for induction of interferon-beta (IFN-beta) and cellular antiviral response. J. Biol. Chem..

[14] Senftleben U (2001). Activation by IKKalpha of a second, evolutionary conserved, NF-kappa B signaling pathway. Science.

[15] Varfolomeev E (2007). IAP antagonists induce autoubiquitination of c-IAPs, NF-kappaB activation, and TNFalpha-dependent apoptosis. Cell.

[16] Xiao G, Harhaj EW, Sun SC (2001). NF-kappaB-inducing kinase regulates the processing of NF-kappaB2 p100. Mol. Cell.

[17] Parvatiyar K (2012). The helicase DDX41 recognizes the bacterial secondary messengers cyclic di-GMP and cyclic di-AMP to activate a type I interferon immune response. Nat. Immunol..

[18] Woodward JJ, Iavarone AT, Portnoy DA (2010). c-di-AMP secreted by intracellular Listeria monocytogenes activates a host type I interferon response. Science.

[19] Jin J (2014). Noncanonical NF-κB pathway controls the production of type I interferons in antiviral innate immunity. Immunity.

[20] Sun L, Wu J, Du F, Chen X, Chen ZJ (2013). Cyclic GMP-AMP synthase is a cytosolic DNA sensor that activates the type I interferon pathway. Science.

[21] Zhou Q (2014). The ER-associated protein ZDHHC1 is a positive regulator of DNA virus-triggered, MITA/STING-dependent innate immune signaling. Cell Host Microbe.

[22] Ishikawa H, Ma Z, Barber GN (2009). STING regulates intracellular DNA-mediated, type I interferon-dependent innate immunity. Nature.

[23] Ouyang S (2012). Structural analysis of the STING adaptor protein reveals a hydrophobic dimer interface and mode of cyclic di-GMP binding. Immunity.

[24] Sun W (2009). ERIS, an endoplasmic reticulum IFN stimulator, activates innate immune signaling through dimerization. Proc. Natl. Acad. Sci. USA.

[25] Tsuchida T (2010). The ubiquitin ligase TRIM56 regulates innate immune responses to intracellular double-stranded DNA. Immunity.

[26] Li Z (2015). PPM1A regulates antiviral signaling by antagonizing TBK1-mediated STING phosphorylation and aggregation. PLoS Pathog..

[27] Tanaka Y, Chen ZJ (2012). STING specifies IRF3 phosphorylation by TBK1 in the cytosolic DNA signaling pathway. Sci. Signal..

[28] Vandenabeele P, Bertrand MJM (2012). The role of the IAP E3 ubiquitin ligases in regulating pattern-recognition receptor signalling. Nat. Rev. Immunol..

[29] Yang XD, Sun SC (2015). Targeting signaling factors for degradation, an emerging mechanism for TRAF functions. Immunol. Rev..

[30] Saitoh T (2009). Atg9a controls dsDNA-driven dynamic translocation of STING and the innate immune response. Proc. Natl. Acad. Sci. USA.

[31] Dobbs N (2015). STING activation by translocation from the ER is associated with infection and autoinflammatory disease. Cell. Host. Microbe.

[32] Liu S (2015). Phosphorylation of innate immune adaptor proteins MAVS, STING, and TRIF induces IRF3 activation. Science.

[33] Zhong B (2009). The ubiquitin ligase RNF5 regulates antiviral responses by mediating degradation of the adaptor protein MITA. Immunity.

[34] Wang Y (2015). TRIM30α is a negative-feedback regulator of the intracellular DNA and DNA virus-triggered response by targeting STING. PLoS Pathog..

[35] Xing J (2017). TRIM29 promotes DNA virus infections by inhibiting innate immune response. Nat. Commun..

[36] Luo WW (2016). iRhom2 is essential for innate immunity to DNA viruses by mediating trafficking and stability of the adaptor STING. Nat. Immunol..

[37] Ling L, Cao Z, Goeddel DV (1998). NF-kappaB-inducing kinase activates IKK-alpha by phosphorylation of Ser-176. Proc. Natl. Acad. Sci. USA.

[38] Razani B, Reichardt AD, Cheng G (2011). Non-canonical NF-κB signaling activation and regulation: principles and perspectives. Immunol. Rev..

[39] Régnier CH (1997). Identification and characterization of an IkappaB kinase. Cell.

[40] Sun SC (2012). The noncanonical NF-κB pathway. Immunol. Rev..

[41] Jiang X (2012). Ubiquitin-induced oligomerization of the RNA sensors RIG-I and MDA5 activates antiviral innate immune response. Immunity.

[42] Li X (2013). Cyclic GMP-AMP synthase is activated by double-stranded DNA-induced oligomerization. Immunity.

[43] Peisley A, Wu B, Xu H, Chen ZJ, Hur S (2014). Structural basis for ubiquitin-mediated antiviral signal activation by RIG-I. Nature.

[44] Zhang X (2014). The cytosolic DNA sensor cGAS forms an oligomeric complex with DNA and undergoes switch-like conformational changes in the activation loop. Cell Rep..

[45] Hou F (2011). MAVS forms functional prion-like aggregates to activate and propagate antiviral innate immune response. Cell.

[46] Liu B (2017). The ubiquitin E3 ligase TRIM31 promotes aggregation and activation of the signaling adaptor MAVS through Lys63-linked polyubiquitination. Nat. Immunol..

[47] Fitzgerald KA (2003). IKKepsilon and TBK1 are essential components of the IRF3 signaling pathway. Nat. Immunol..

[48] Larabi A (2013). Crystal structure and mechanism of activation of TANK-binding kinase 1. Cell Rep..

[49] Ma X (2012). Molecular basis of Tank-binding kinase 1 activation by transautophosphorylation. Proc. Natl. Acad. Sci. USA.

[50] Mori M (2004). Identification of Ser-386 of interferon regulatory factor 3 as critical target for inducible phosphorylation that determines activation. J. Biol. Chem..

[51] Sharma S (2003). Triggering the interferon antiviral response through an IKK-related pathway. Science.

[52] Lin X (1998). Molecular determinants of NF-kappaB-inducing kinase action. Mol. Cell. Biol..

[53] Nakhaei P (2009). The E3 ubiquitin ligase Triad3A negatively regulates the RIG-I/MAVS signaling pathway by targeting TRAF3 for degradation. PLoS. Pathog..

[54] Zhang M (2016). USP18 recruits USP20 to promote innate antiviral response through deubiquitinating STING/MITA. Cell Res..

[55] Luo WW (2017). iRhom2 is essential for innate immunity to RNA virus by antagonizing ER- and mitochondria-associated degradation of VISA. PLoS Pathog..

[56] Gray CM, McCorkell KA, Chunduru SK, McKinlay MA, May MJ (2014). Negative feedback regulation of NF-κB-inducing kinase is proteasome-dependent but does not require cellular inhibitors of apoptosis. Biochem. Biophys. Res. Commun..

[57] Razani B (2010). Negative feedback in noncanonical NF-kappaB signaling modulates NIK stability through IKKalpha-mediated phosphorylation. Sci. Signal..

[58] Ishii KJ (2008). TANK-binding kinase-1 delineates innate and adaptive immune responses to DNA vaccines. Nature.

[59] Miyahira AK, Shahangian A, Hwang S, Sun R, Cheng G (2009). TANK-binding kinase-1 plays an important role during in vitro and in vivo type I IFN responses to DNA virus infections. J. Immunol..

[60] Jin J (2012). The kinase TBK1 controls IgA class switching by negatively regulating noncanonical NF-κB signaling. Nat. Immunol..

[61] Sohn J, Hur S (2016). Filament assemblies in foreign nucleic acid sensors. Curr. Opin. Struct. Biol..

[62] Vajjhala, P. R., Ve, T., Bentham, A., Stacey, K. J. & Kobe, B. The molecular mechanisms of signaling by cooperative assembly formation in innate immunity pathways. *Mol. Immunol*. 10.1016/j.molimm.2017.02.012 (2017). 10.1016/j.molimm.2017.02.012 28249680

[63] Junt T, Barchet W (2015). Translating nucleic acid-sensing pathways into therapies. Nat. Rev. Immunol..

[64] McFadden G, Mohamed MR, Rahman MM, Bartee E (2009). Cytokine determinants of viral tropism. Nat. Rev. Immunol..

[65] Benedict CA (2001). Lymphotoxins and cytomegalovirus cooperatively induce interferon-beta, establishing host-virus détente. Immunity.

[66] He JQ (2006). Rescue of TRAF3-null mice by p100 NF-kappa B deficiency. J. Exp. Med..

[67] Xu Y, Cheng G, Baltimore D (1996). Targeted disruption of TRAF3 leads to postnatal lethality and defective T-dependent immune responses. Immunity.

[68] Parvatiyar K, Barber GN, Harhaj EW (2010). TAX1BP1 and A20 inhibit antiviral signaling by targeting TBK1-IKKi kinases. J. Biol. Chem..

[69] Schindelin J (2012). Fiji: an open-source platform for biological-image analysis. Nat. Methods.

