# Supplementary material for: A TRAF3-NIK module differentially regulates DNA vs RNA pathways in innate immune signaling
Source: Nat Commun. 2018 Jul 17;9:2770. doi: 10.1038/s41467-018-05168-7 (PMC6050272; doi:10.1038/s41467-018-05168-7)
Supplement: Supplementary file 1 — Supplementary Information [file 41467_2018_5168_MOESM1_ESM.pdf]

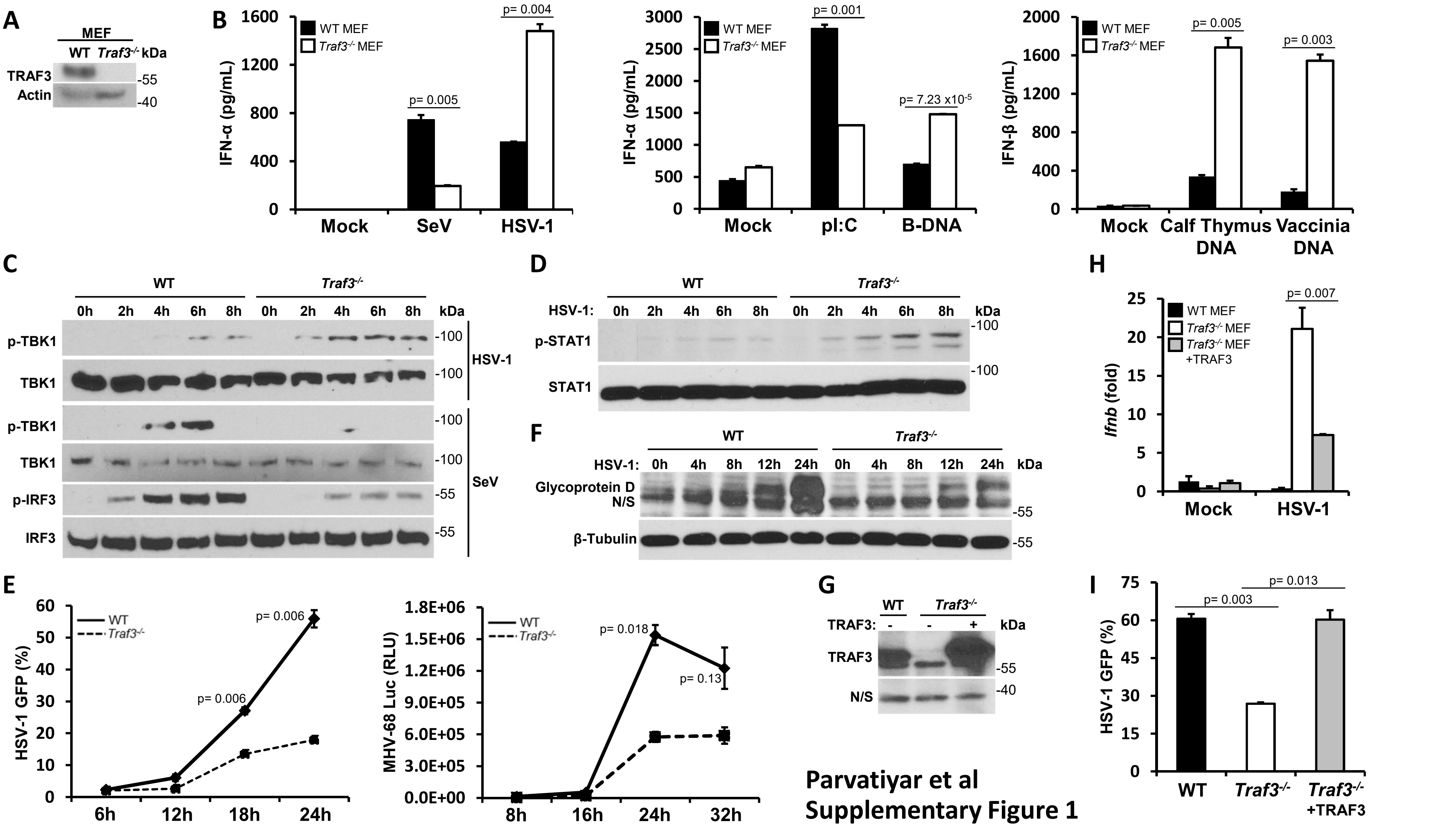

Parvatiyar et al
Supplementary Figure 1

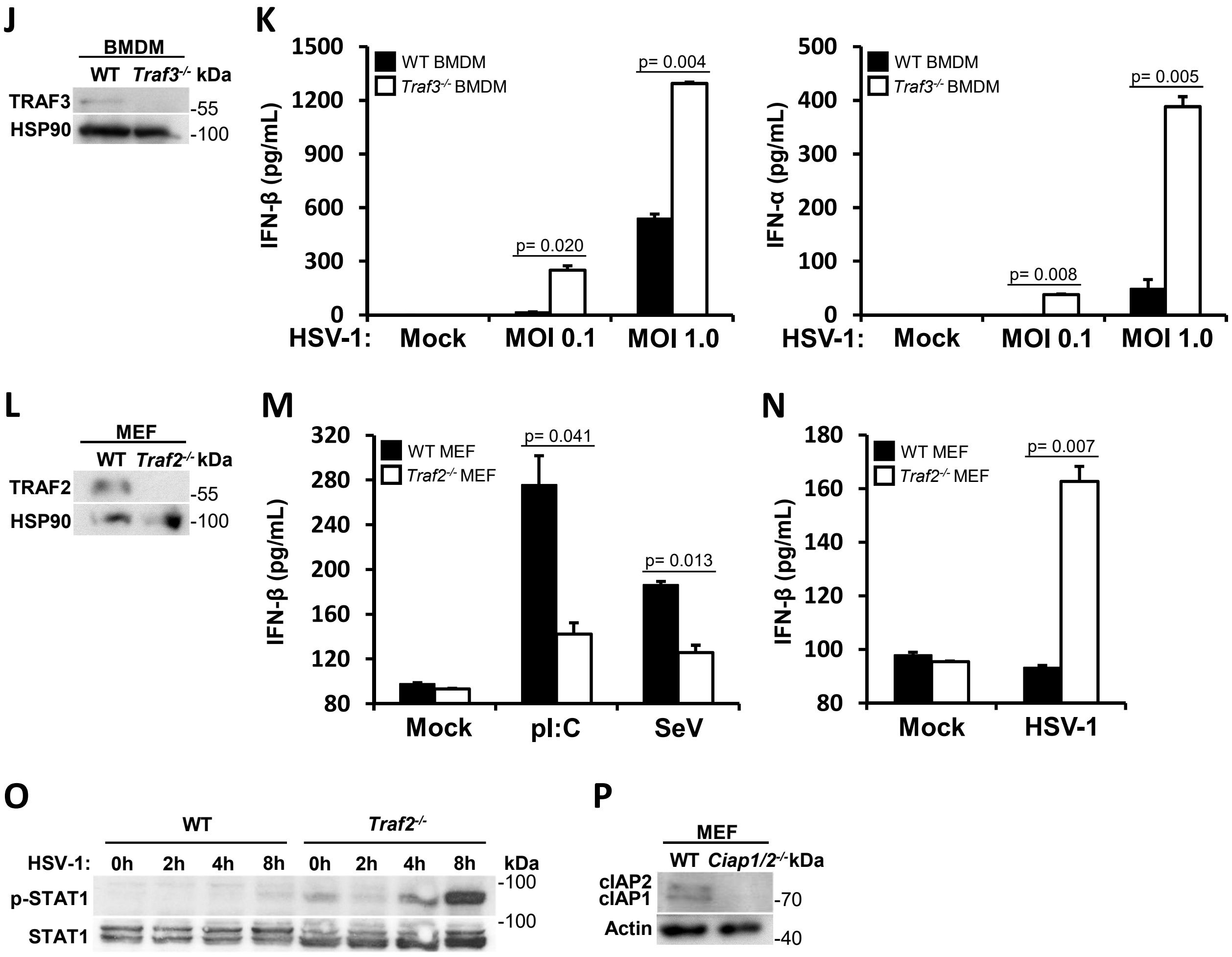

Parvatiyar et al

Supplementary Figure 1 Continued

Supplementary Figure 1. Alternative NF-κB pathway regulators play an inhibitory role in the DNA pathway.

(A) Immunoblot analysis of TRAF3 in WT and *Traf3*$^{-/-}$ MEF cells. Actin serves as a loading control. Results are representative of two independent experiments.

(B) ELISA for IFN-α4 or IFN-β cytokines from supernatants collected 24 hours post infection or stimulation of WT and *Traf3*$^{-/-}$ MEFs infected with Sendai virus (SeV) or HSV-1 (MOI 0.1) (left panel), transfected with poly (I:C) (500 ng/mL) or B-DNA (500 ng/mL) (center panel), or transfected with calf thymus DNA (5 µg/mL) or Vaccinia DNA (50 ng/mL) (right panel). Data are means ± SEM of one experiment run in duplicates out of two independent experiments.

(C) Immunoblot analysis of TBK1 or IRF3 activation in WT and *Traf3*$^{-/-}$ MEF cells infected HSV-1 (MOI 0.1) (upper panels) or SeV (MOI 0.1) (lower panels) at the indicated time points. Results are representative of two independent experiments.

(D) Immunoblot analysis of STAT1 activation in WT and *Traf3*$^{-/-}$ MEF cells infected HSV-1 (MOI 0.1) at the indicated time points. Results are representative of three independent experiments.

(E) Viral load of HSV-1 or MHV-68 in WT and *Traf3*$^{-/-}$ MEFs infected with HSV-1 GFP (MOI 0.1) (left panel) or MHV-68-luciferase (MOI 0.1) (right panel) at the indicated time points. Data are means ± SEM of one experiment run in duplicates out of 2-3 independent experiments.

(F) Immunoblot analysis of HSV-1 encoded protein Glycoprotein D in WT and *Traf3*$^{-/-}$ MEF cells infected HSV-1 (MOI 0.1) at the indicated time points. β-Tubulin serves as a loading control. N/S, non-specific. Results are representative of two independent experiments.

(G) Immunoblot analysis of TRAF3 expression in WT MEF cells or in *Traf3*$^{-/-}$ MEF cells reconstituted with empty vector or a pBABE TRAF3 plasmid construct (N/S, non-specific). Results are representative of two independent experiments.

(H) Q-PCR of IFN-β mRNA in MEF cells from (G) infected with HSV-1 (MOI 0.1) for 8 hours. Data are means ± SEM of one experiment run in triplicates out of two independent experiments.

(I) Population of HSV-1 in MEF cells from (G) infected with HSV-1 GFP measured 24 hours post infection. Data are means ± SEM of one experiment run in duplicates out of two independent experiments.

(J) Immunoblot analysis of TRAF3 in WT and *Traf3*$^{-/-}$ BMDMs. HSP90 serves as a loading control. Results are representative of two independent experiments.

(K) ELISA for IFN-β (left panel) or IFN-α4 (right panel) from supernatants of WT and *Traf3*$^{-/-}$ BMDMs collected 24 hours after infection with HSV-1 at the indicated MOIs. Data are means ± SEM of one experiment run in duplicates out of two independent experiments.

(L) Immunoblot analysis of TRAF2 in WT and *Traf2*$^{-/-}$ MEF cells. HSP90 serves as a loading control. Results are representative of two independent experiments.

(M-N) IFN-β ELISAs from supernatants taken 24 hours after poly (I:C) transfection (100 ng/mL) or SeV infection (MOI 0.1) (M) or HSV-1 infection (MOI 0.1) (N) in WT and *Traf2*$^{-/-}$ MEF cells. Data are means ± SEM of one experiment run in duplicates out of two independent experiments.

(O) Immunoblot analysis of STAT1 activation in WT and *Traf2*$^{-/-}$ MEFs infected HSV-1 (MOI 0.1) at the indicated time points. Results are representative of two independent experiments.

(P) Immunoblot analysis of cIAP1 and cIAP2 in WT and *Ciap1/2*$^{-/-}$ MEF cells. Actin serves as a loading control. Results are representative of two independent experiments.

$p < 0.05$ is considered significant.

Parvatiyar et al
Supplementary Figure 1

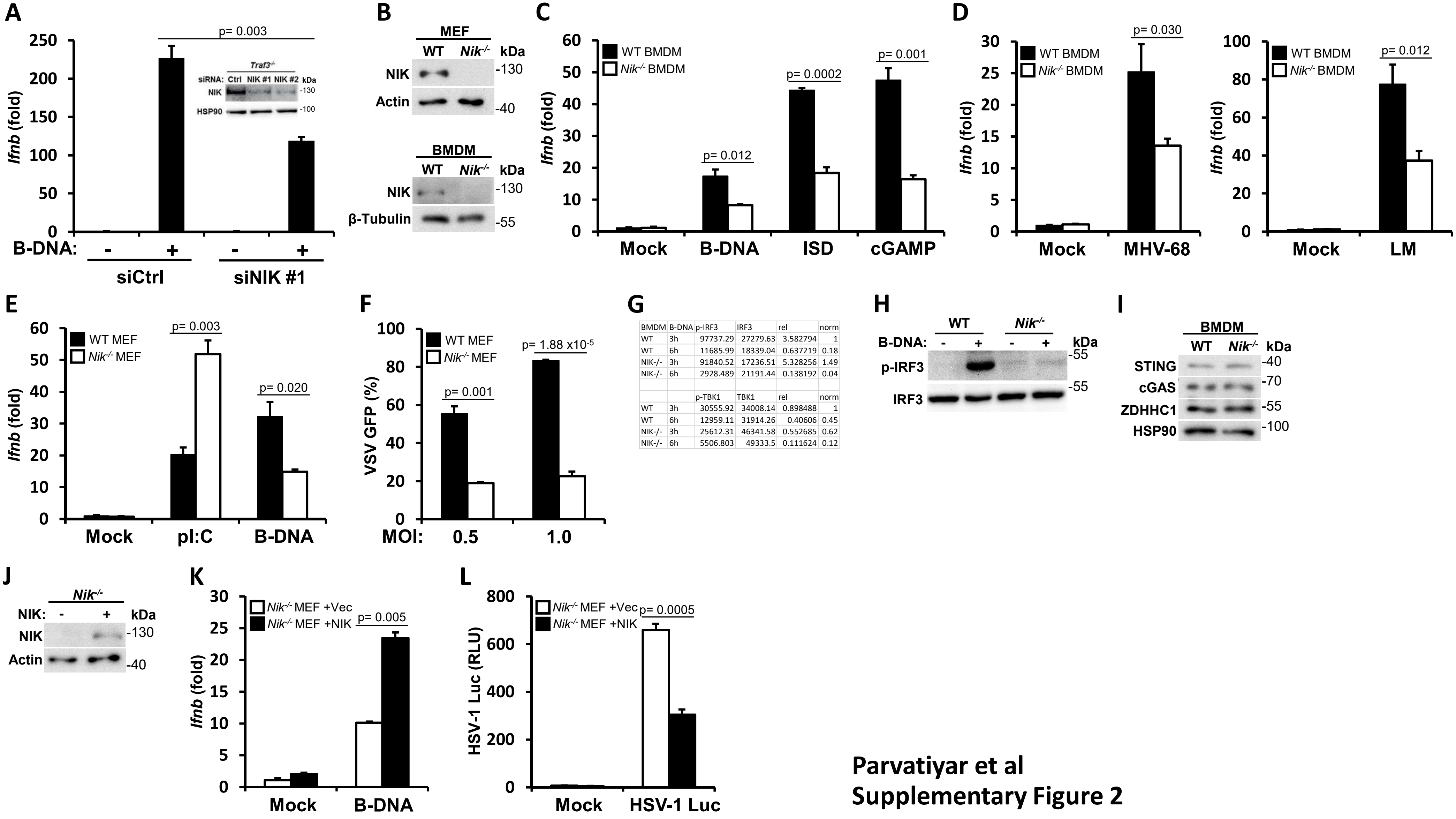

BMDM	B-DNA	p-IRF3	IRF3	rel	norm
WT	3h	97737.29	27279.63	3.582794	1
WT	6h	11685.99	18339.04	0.637219	0.18
NIK-/-	3h	91840.52	17236.51	5.328256	1.49
NIK-/-	6h	2928.489	21191.44	0.138192	0.04
		p-TBK1	TBK1	rel	norm
WT	3h	30555.92	34008.14	0.898488	1
WT	6h	12959.11	31914.26	0.40606	0.45
NIK-/-	3h	25612.31	46341.58	0.552685	0.62
NIK-/-	6h	5506.803	49333.5	0.111624	0.12

Parvatiyar et al
Supplementary Figure 2

Supplementary Figure 2. NIK plays a positive regulatory role in the DNA pathway.

(A) Q-PCR for IFN-β mRNA induction in *Traf3*$^{-/-}$ MEFs treated with control siRNA or siRNA targeting NIK (100 pmol) followed by B-DNA transfection (100 ng/mL) for 4 hours. Data are means ± SEM of one experiment run in triplicates out of two independent experiments. Inset: Immunoblot analysis of NIK expression in *Traf3*$^{-/-}$ MEFs treated with control siRNA or siRNA targeting NIK (100 pmol). HSP90 serves as a loading control. Results are representative of two independent experiments.

(B) Immunoblot analysis of NIK in WT and *Nik*$^{-/-}$ MEFs (upper panel) or BMDMs (lower panel) pre-treated with MG132 (10 µM). Actin and β-Tubulin serve as loading controls. Results are representative of two independent experiments.

(C) Q-PCR for IFN-β mRNA in WT and *Nik*$^{-/-}$ BMDMs transfected with B-DNA (100 ng/mL), immunostimulatory DNA (ISD) (200 ng/mL), or cyclic GMP-AMP (cGAMP) (200 ng/mL) for 8 hours. Data are means ± SEM of one experiment run in triplicates out of two independent experiments.

(D) Q-PCR of IFN-β mRNA levels in WT and *Nik*$^{-/-}$ BMDMs infected with MHV-68 (MOI 1.0) (left panel) or with *Listeria monocytogenes* (LM) (MOI 1.0) (right panel) for 24 hours. Data are means ± SEM of one experiment run in triplicates out of two independent experiments.

(E) Q-PCR for IFN-β mRNA in WT and *Nik*$^{-/-}$ MEFs transfected with poly (I:C) (500 ng/mL) or B-DNA (100 ng/mL) for 4 hours. Data are means ± SEM of one experiment run in triplicates out of two independent experiments.

(F) Population of vesicular stomatitis virus (VSV) in WT and *Nik*$^{-/-}$ MEFs infected with VSV GFP measured 24 hours post infection at the indicated MOIs. Data are means ± SEM of one experiment run in triplicates out of two independent experiments.

(G) Immunoblot image density ratio between p-IRF3/IRF3 and p-TBK1/TBK1 from Figure 2G using ImageJ software.

(H) Immunoblot analysis of IRF3 activation in WT and *Nik*$^{-/-}$ MEFs transfected with B-DNA (2 µg/mL) for 4 hours. Results are representative of two independent experiments.

(I) Immunoblot analysis of STING, cGAS, and ZDHHC1 in WT and *Nik*$^{-/-}$ BMDMs. Results are representative of three independent experiments.

(J) Immunoblot analysis of NIK expression in WT MEF cells or in *Nik*$^{-/-}$ MEF cells reconstituted with empty vector or a pBABE NIK plasmid construct. Actin serves as a loading control. Results are representative of two independent experiments.

(K) Q-PCR of IFN-β mRNA in MEF cells from (J) transfected with B-DNA (500 ng/mL) for 4 hours. Data are means ± SEM of one experiment run in duplicates out of two independent experiments.

(L) Viral loads of HSV-1 encoding luciferase in MEF cells from (J) infected with HSV-1 Luc (MOI 0.1) measured 24 hours post infection. Data are means ± SEM of one experiment run in triplicates out of two independent experiments.

$p < 0.05$ is considered significant.

Parvatiyar et al
Supplementary Figure 2

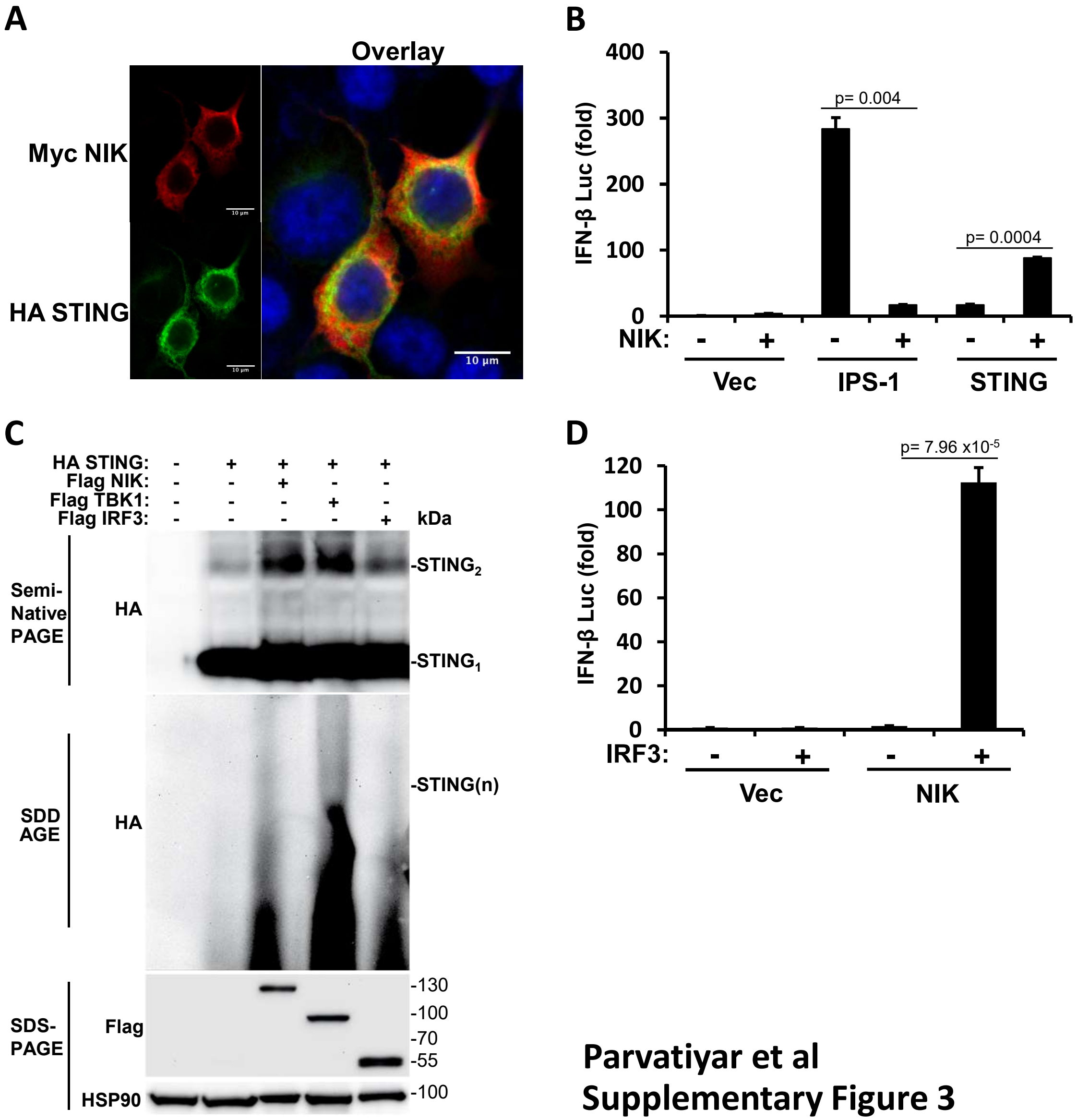

Parvatiyar et al
Supplementary Figure 3

Supplementary Figure 3. NIK enhances STING activity.

(A) Confocal microscopy of HEK 293T cells transfected with Myc-NIK and HA-STING expression constructs. DNA was stained with DAPI (shown in blue). Results are representative of two independent experiments. Scale bar represents 10 µm.

(B) IFN-β luciferase reporter assay in HEK 293T cells co-transfected with NIK and STING or IPS-1 encoding plasmids. Data are means ± SEM of one experiment run in duplicates out of three independent experiments.

(C) Semi-native PAGE and SDD AGE immunoblot analysis of STING dimerization and aggregation in HEK 293T cells co-transfected with HA-STING and Flag-NIK, TBK1, or IRF3 encoding plasmids. Results are representative of two independent experiments.

(D) IFN-β luciferase reporter assay in HEK 293T cells co-transfected with plasmids encoding IRF3 and NIK. Data are means ± SEM of one experiment run in triplicates out of three independent experiments.

$p < 0.05$ is considered significant.

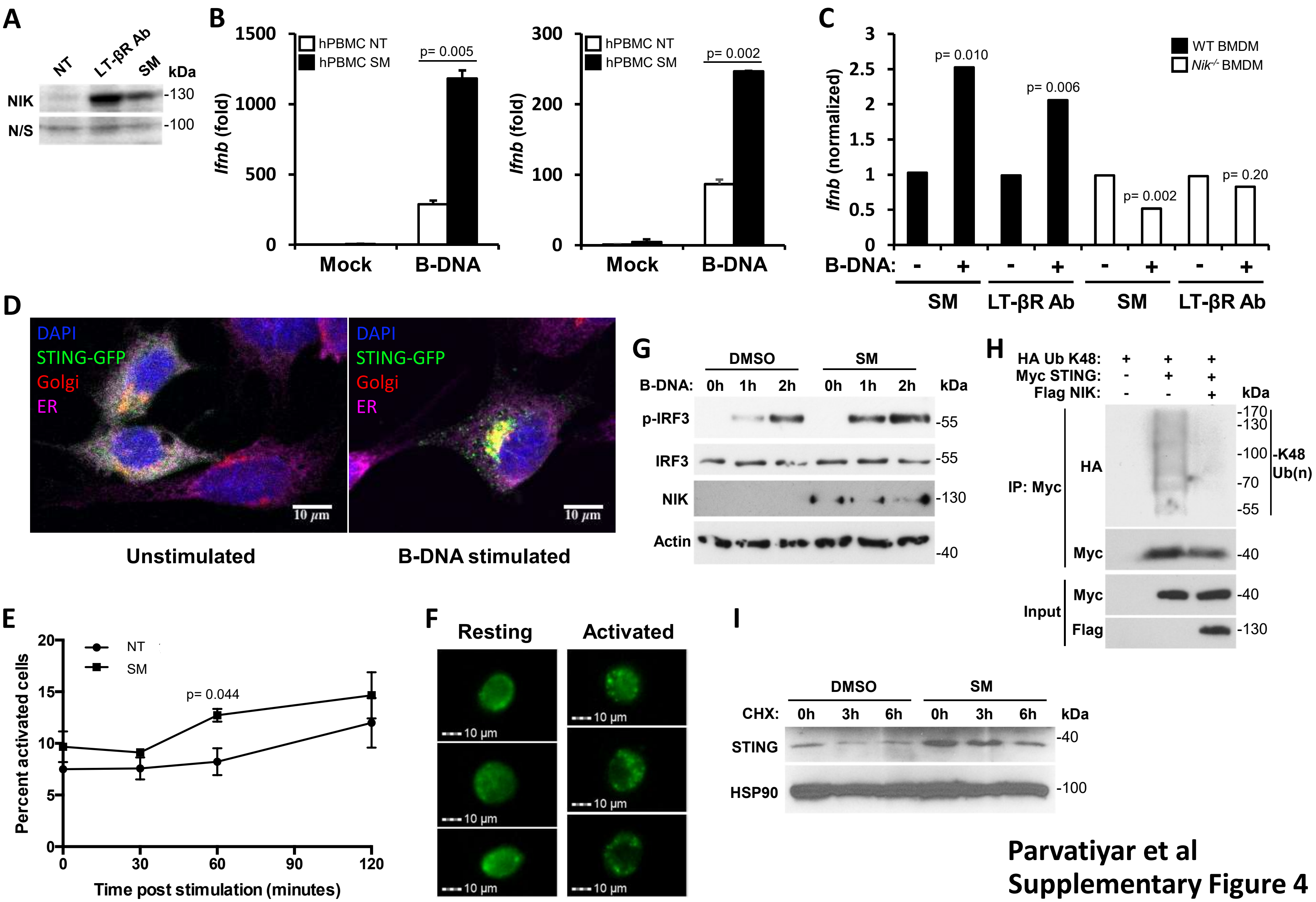

Parvatiyar et al
Supplementary Figure 4

Supplementary Figure 4. Pharmacological activation of NIK increases STING signaling.

(A) Immunoblot analysis for NIK expression in WT MEF cells stimulated with DMSO control (NT), LT-βR agonistic antibody (2 μg/mL), or with SMAC mimetic (SM) (1 μg/mL) for 12 hours. N/S, non-specific. Results are representative of two independent experiments.

(B) Q-PCR of IFN-β mRNA levels in human PBMCs (donor #2 left panel, donor #3 right panel) pre-treated with DMSO (NT) or SM (1 μg/mL) for 16 hours followed by B-DNA transfection (100 ng/mL) for 4 hours. Data are means ± SEM of experiments run in duplicates and represent two out of three independent experiments where the third independent experiment (donor #1) is presented in Figure 4E.

(C) Q-PCR for IFN-β mRNA (normalized to DMSO treated) in WT or *Nik*$^{-/-}$ BMDMs pre-treated with SM (1 μg/mL) or LT-βR agonistic antibody (2 μg/mL) for 16 hours, followed by B-DNA transfection (100 ng/mL) for 6 hours. Data are means ± SEM of one experiment run in triplicates out of two independent experiments.

(D) Confocal imaging of STING trafficking in *Sting*$^{-/-}$ MEFs stably reconstituted with STING-GFP. Cells were unstimulated or transfected with B-DNA (500 ng/mL) for 2h as indicated. The cells were then stained with DAPI (DNA), anti-calnexin (ER) and anti-GM130 (Golgi) antibodies while localization of STING-GFP was determined by confocal microscopy. Results are representative of two independent experiments. Scale bar represents 10 μm.

(E-F) Imaging flow cytometric analysis of STING puncta formation in *Sting*$^{-/-}$ MEFs stably expressing STING-GFP (described in S4D) that were pre-treated with SM (1 μg/mL) for 16 hours followed by B-DNA transfection (500 ng/mL) for 0, 30, 60, or 120 minutes (E). Cells were gated based on puncta formation and the percentage of activated cells was compared between DMSO (NT) and SM treated cells at all timepoints. Data represent the means ± SEM of two independent experiments. Representative examples of what are determined to be resting and activated cells to calculate % activation values in S4E (F). Scale bar represents 10 μm.

(G) Immunoblot analysis for IRF3 activation in WT MEF cells pre-treated with vehicle control (DMSO) or SM (1 μg/mL) for 16 hours followed by B-DNA transfection (2 μg/mL) at the time points indicated. Results are representative of two independent experiments.

(H) Immunoprecipitation and immunoblot analysis of HA-K48-linked ubiquitin chains covalently attached to Myc-STING in the absence or presence of Flag-NIK co-transfected in HEK 293T cells. Results are representative of two independent experiments.

(I) Immunoblot analysis of STING stability in A549 cells pre-treated with vehicle control (DMSO) or SM (1 μg/mL) for 16 hours followed by B-DNA transfection (2 μg/mL) for 2 hours, then treated with cycloheximide (CHX, 100 μg/mL) for the indicated time points. HSP90 serves as a loading control. Results are representative of two independent experiments.

$p < 0.05$ is considered significant.

Parvatiyar et al
Supplementary Figure 4

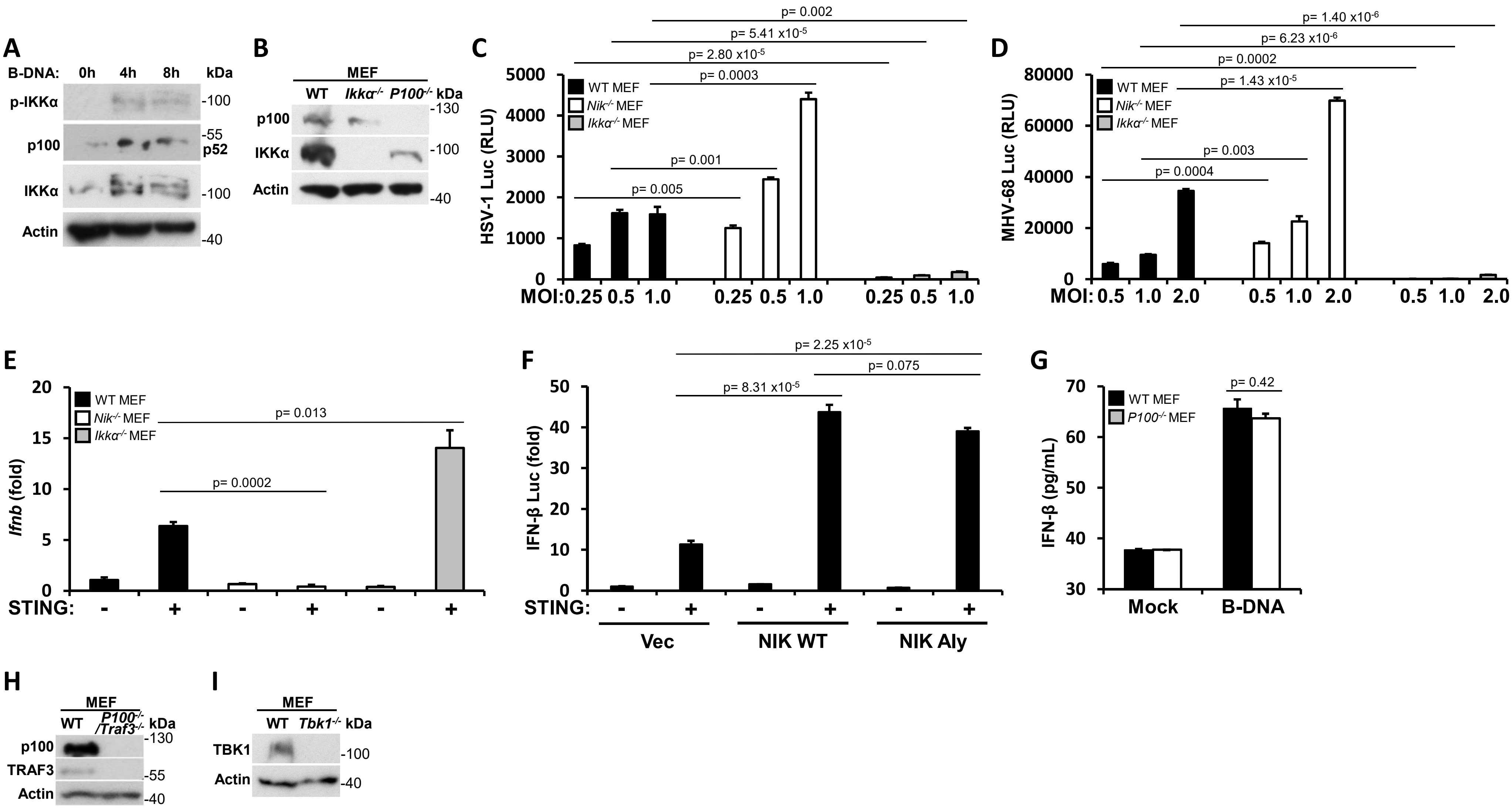

Parvatiyar et al
Supplementary Figure 5

Supplementary Figure 5. NIK enhances DNA pathway activation independently of alternative NF-κB components.

(A) Immunoblot analysis of IKKα activation and p52 accumulation in A549 cells transfected with B-DNA (2 µg/mL) for the indicated time points. Results are representative of two independent experiments.

(B) Immunoblot analysis of IKKα and p100 expression in WT, *P100*$^{-/-}$, and *Ikkα*$^{-/-}$ MEFs. Actin serves as a loading control. Results are representative of two independent experiments.

(C-D) Viral load in WT, *Nik*$^{-/-}$, and *Ikkα*$^{-/-}$ MEFs infected with HSV-1 luciferase (A) or MHV-68 luciferase (B) 24 hours post infection at the indicated MOIs. Data are means ± SEM of one experiment run in triplicates out of two independent experiments.

(E) Q-PCR for IFN-β mRNA levels in WT, *Nik*$^{-/-}$, and *Ikkα*$^{-/-}$ MEFs transfected with STING plasmid for 24 hours. Data are means ± SEM of one experiment run in triplicates out of two independent experiments.

(F) IFN-β luciferase reporter assay in HEK 293T cells co-transfected with plasmids encoding STING and wild type NIK, (NIK WT), or a NIK mutant that does not interact with IKKα (NIK Aly). Data are means ± SEM of one experiment run in triplicates out of three independent experiments.

(G) ELISA for IFN-β from supernatants of WT and *P100*$^{-/-}$ MEFs collected 24 hours after B-DNA transfection (500 ng/mL). Data are means ± SEM of one experiment run in duplicates out of two independent experiments.

(H) Immunoblot analysis of p100 and TRAF3 in WT and *P100*$^{-/-}$/*Traf3*$^{-/-}$ MEFs. Actin serves as a loading control. Results are representative of two independent experiments.

(I) Immunoblot analysis of TBK1 expression in WT and *Tbk1*$^{-/-}$ MEFs. Actin serves as a loading control. Results are representative of two independent experiments.

$p < 0.05$ is considered significant.

Parvatiyar et al
Supplementary Figure 5

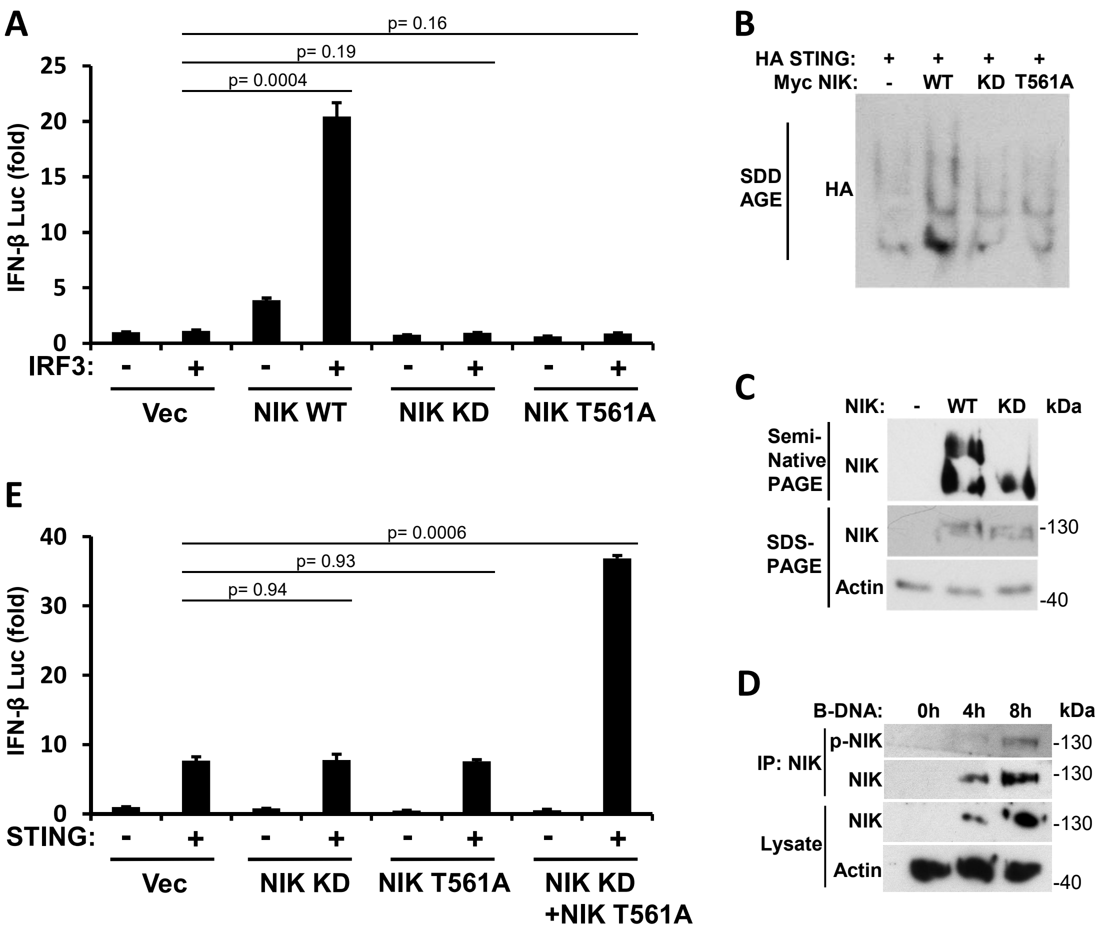

Supplementary Figure 6. NIK requires kinase function and phosphorylation in the DNA pathway

(A) IFN-β luciferase reporter assay in HEK 293T cells co-transfected with plasmids encoding IRF3 and wild type NIK, (NIK WT), kinase dead NIK (NIK KD), or a Thr 561 phospho-acceptor mutant NIK (NIK T561A). Data are means ± SEM of one experiment run in duplicates out of three independent experiments.

(B) SDD AGE immunoblot analysis of STING aggregation in HEK 293T cells co-transfected with HA-STING and Myc-WT or KD NIK plasmids; related to Figure 6B. Results are representative of two independent experiments.

(C) Semi-native PAGE immunoblot analysis of NIK oligomerization in HEK 293T cells transfected with WT or KD NIK plasmids. Results are representative of two independent experiments.

(D) NIK immunoprecipitation and immunoblot analysis of NIK phosphorylation in A549 cells transfected with B-DNA (2 µg/mL) for the indicated time points. Results are representative of two independent experiments.

(E) IFN-β luciferase reporter assay in HEK 293T cells co-transfected with plasmids encoding STING and kinase dead NIK (NIK KD), a Thr 561 phospho-acceptor mutant NIK (NIK T561A), or both NIK KD and NIK T561A together. Data are means ± SEM of one experiment run in duplicates out of two independent experiments.

$p < 0.05$ is considered significant.

Parvatiyar et al
Supplementary Figure 6

A

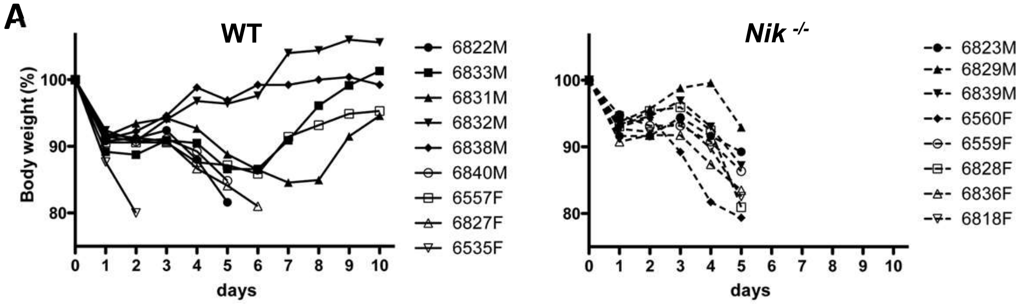

B

#	6822M	6823M	6833M	6829M	6831M	6832M	6838M	6839M	6840M	6560F	6559F	6557F	6827F	6828F	6836F	6535F	6818F
	NIK+/+	NIK-/-	NIK+/+	NIK-/-	NIK+/+	NIK+/+	NIK+/+	NIK-/-	NIK+/+	NIK-/-	NIK-/-	NIK+/+	NIK+/+	NIK-/-	NIK-/-	NIK+/+	NIK-/-
day 0	25	25.1	23.1	25.5	25.9	25	25.8	25.8	27	21.3	24.9	23.4	19.5	17.3	20.6	23.5	20.6
day 1	22.7	23	20.6	23.9	23.7	23.1	23.5	24.1	24.7	19.8	23.6	21.2	17.9	16.1	18.7	20.6	19.1
day 2	22.8	23	20.5	24.4	24.2	22.7	23.8	24.4	24.5	20.1	23.2	21.2	17.8	16.5	18.9	18.8	19
day 3	23.1	23.7	21	25.2	24.4	23.5	24.4	25	24.6	19	23.2	21.2	17.7	16.6	18.9	**X**	19.3
day 4	22	23	20.9	25.4	24	24.2	25.5	24	24.1	17.4	22.6	20.5	16.9	16	18		18.5
day 5	20.4	22.4	20	23.7	23	24.1	25	22.5	22.9	**X** 16.9	21.5	20.4	16.4	**X** 14	**X** 17.2		**X** 17
day 6	**X**	**X**	20	**X**	22.4	24.4	25.6	**X**	**X**		**X**	20.1	15.8				
day 7			21		21.9	26	25.6					21.4	**X**				
day 8			22.2		22	26.1	25.8					21.8					
day 9			22.9		23.7	26.5	25.9					22.2					
day 10			23.4		24.5	26.4	25.6					22.3					

Supplementary Figure 7. NIK plays an essential in vivo role against DNA virus infections.

(A) Percent loss of body weight in WT (n=9) (left panel) or *Nik*$^{-/-}$ (n=8) (right panel) mice infected retro-orbitally with HSV-1 (1 X 10^9 Pfu/mouse); related to Figure 7A.

(B) Body weights of WT and *Nik*$^{-/-}$ mice (in grams) taken for the duration of HSV-1 infection survival curve; related to Figure 7A. X represents animals that either died post infection or were euthanized due to a greater than 20% loss of their initial weight or presented with signs of morbidity.

Original Immunoblots Figure 1

Figure 1C

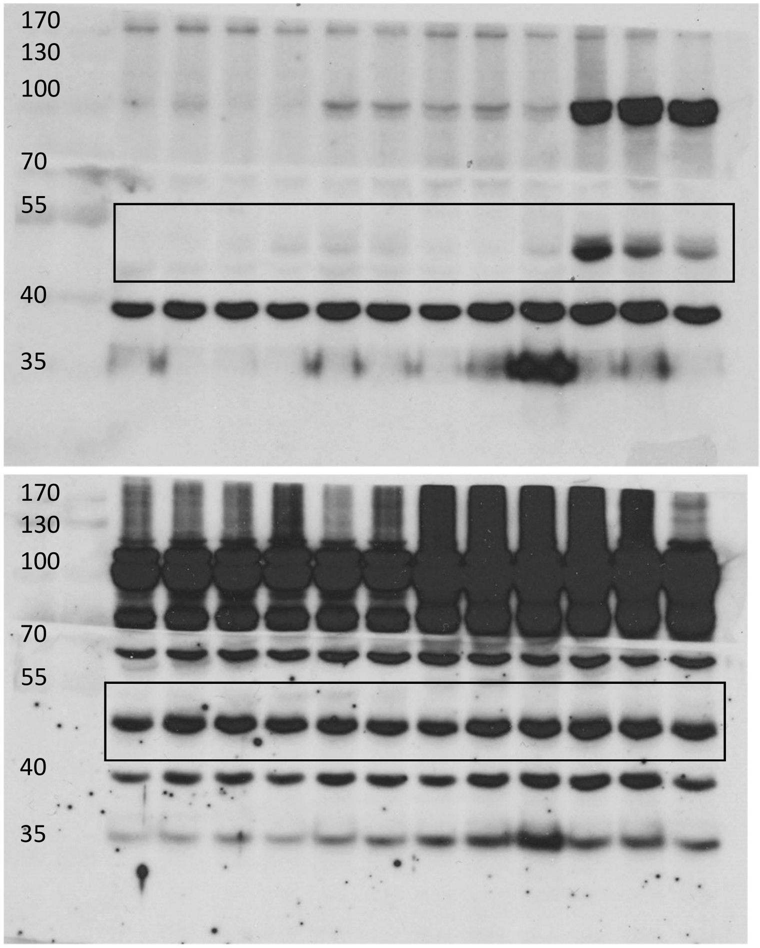

Figure 1F

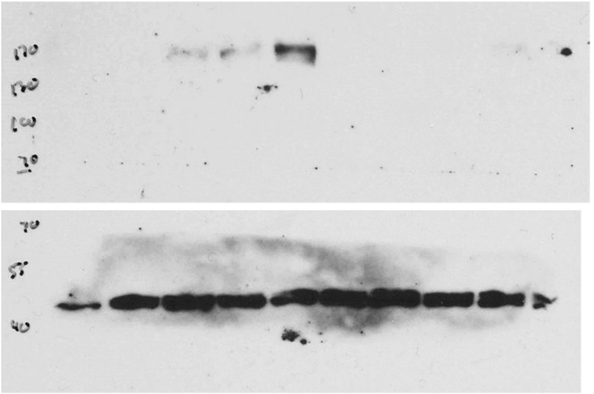

Parvatiyar et al
Supplementary Figure 8

Original Immunoblots Figure 2

Figure 2A, upper

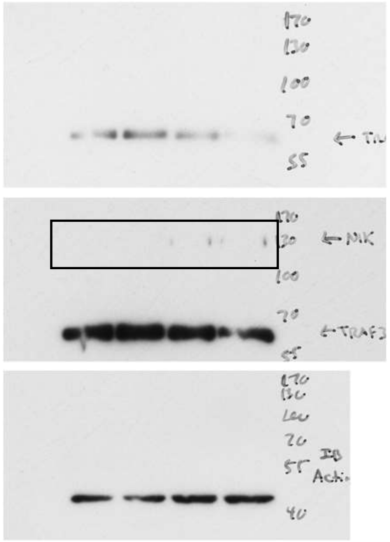

Figure 2A, lower

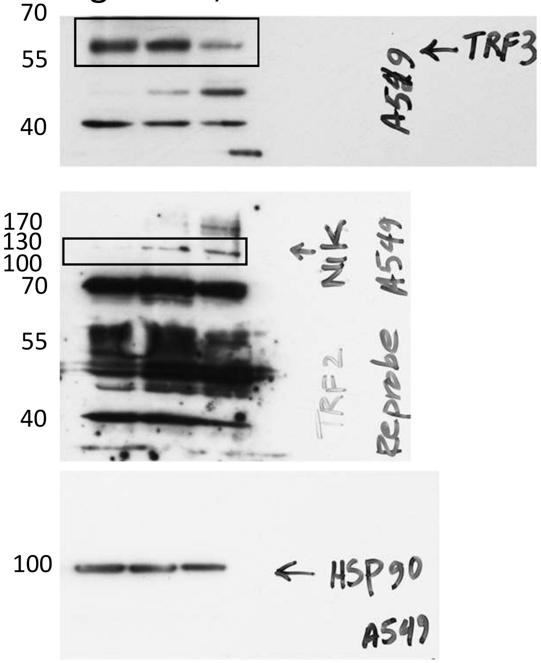

Figure 2G

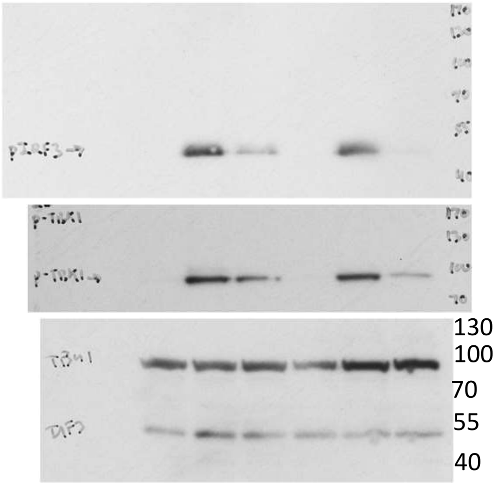

Parvatiyar et al
Supplementary Figure 8

Original Immunoblots Figure 3

Figure 3A

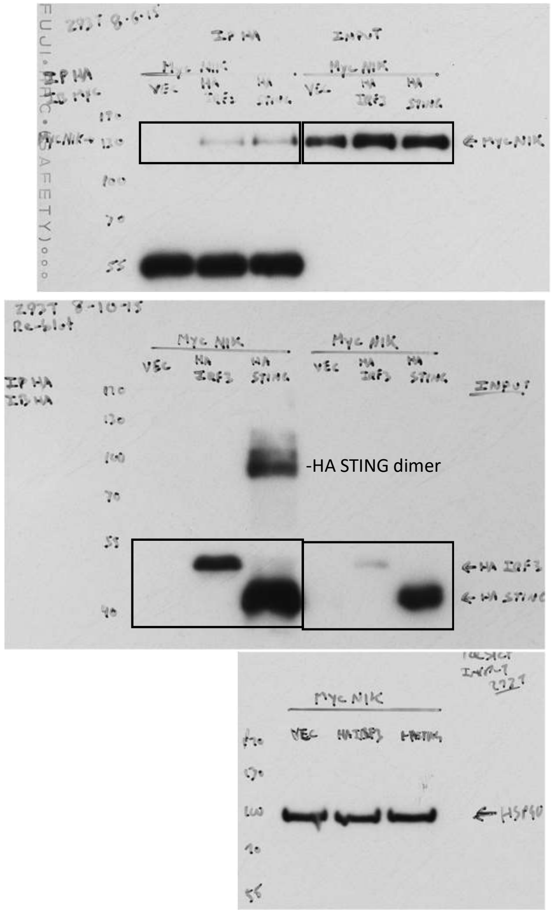

Figure 3B

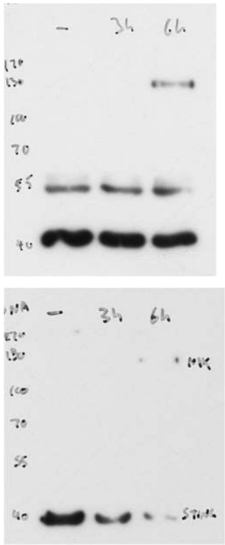

Figure 3D

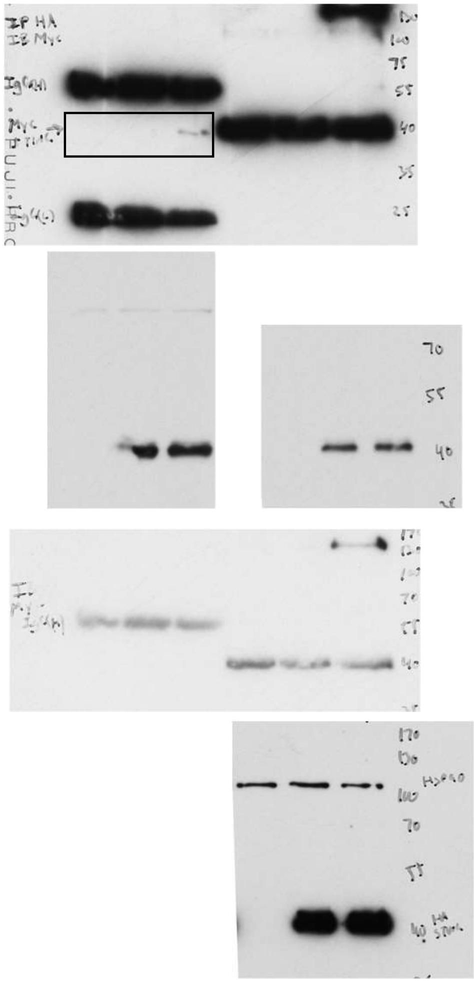

Parvatiyar et al
Supplementary Figure 8

Original Immunoblots Figure 3 Continued

Figure 3E

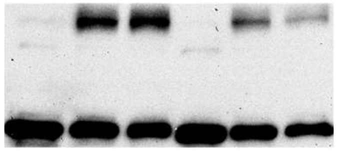

Semi-Native PAGE

Figure 3F

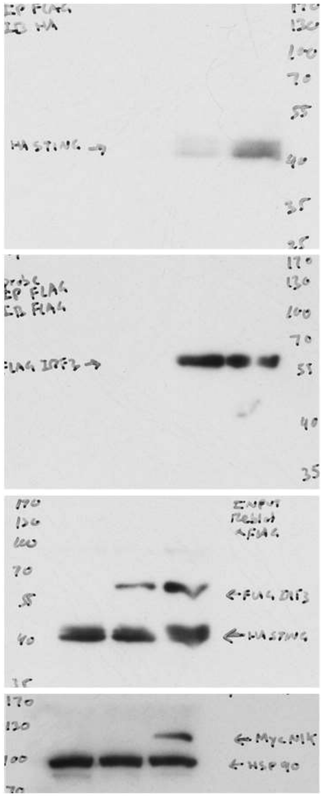

Figure 3G

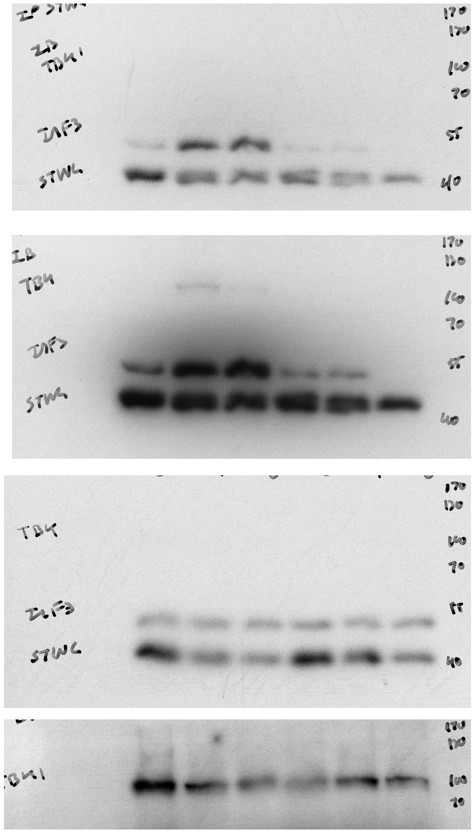

Parvatiyar et al
Supplementary Figure 8

Original Immunoblots Figure 4 and 5

Figure 4G

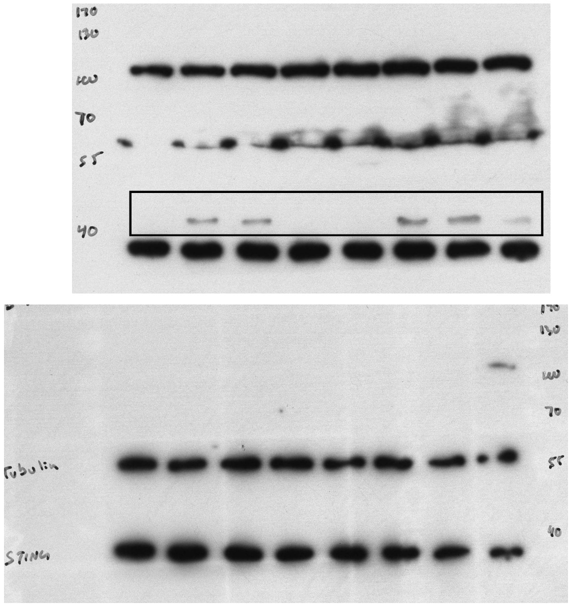

Figure 4H

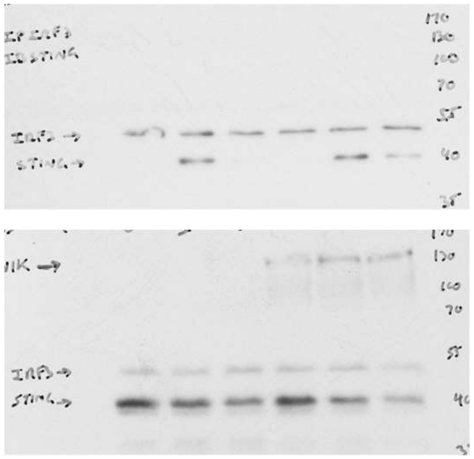

Figure 4I

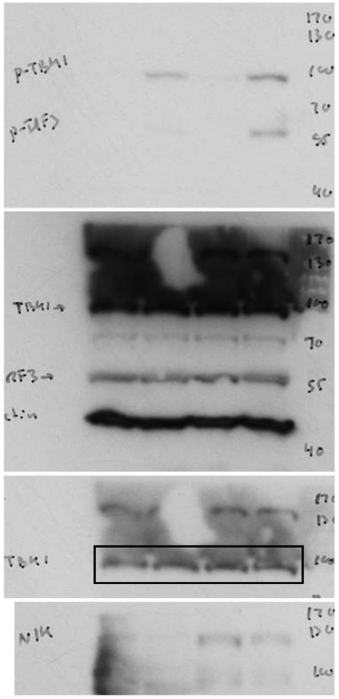

Figure 5G

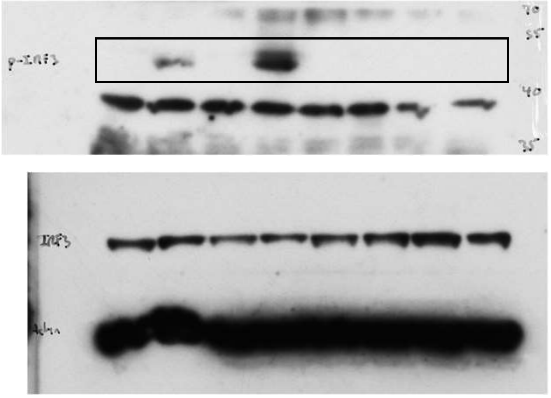

Parvatiyar et al
Supplementary Figure 8

Original Immunoblots Figure 6

Figure 6B

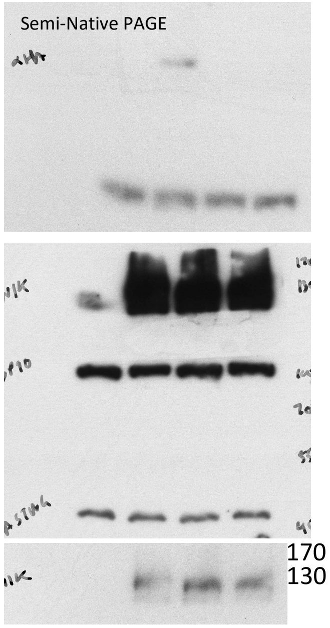

Figure 6C

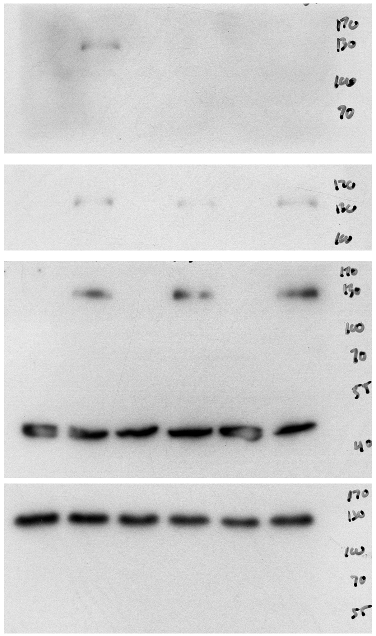

Figure 6D

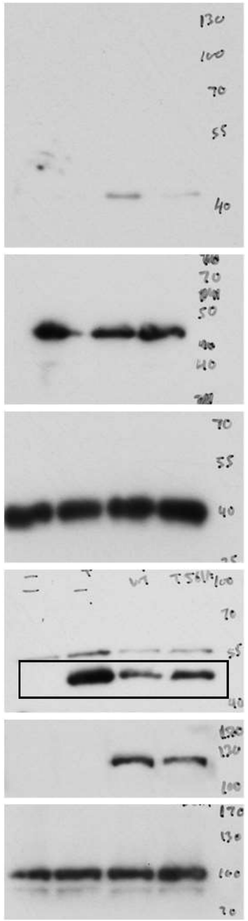

Figure 6E

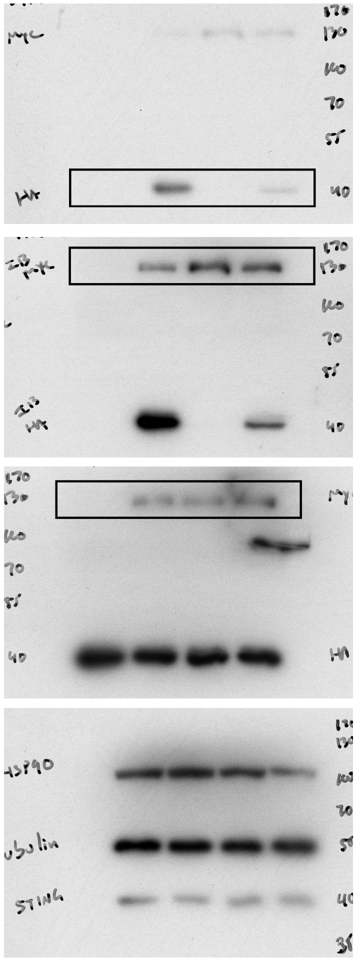

Parvatiyar et al
Supplementary Figure 8

Original Immunoblots Supplementary Figure 1

Supplementary Figure 1A

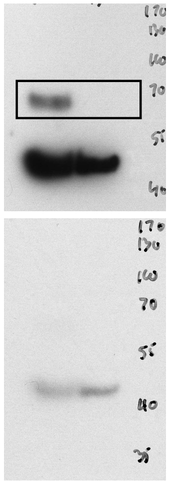

Supplementary Figure 1C

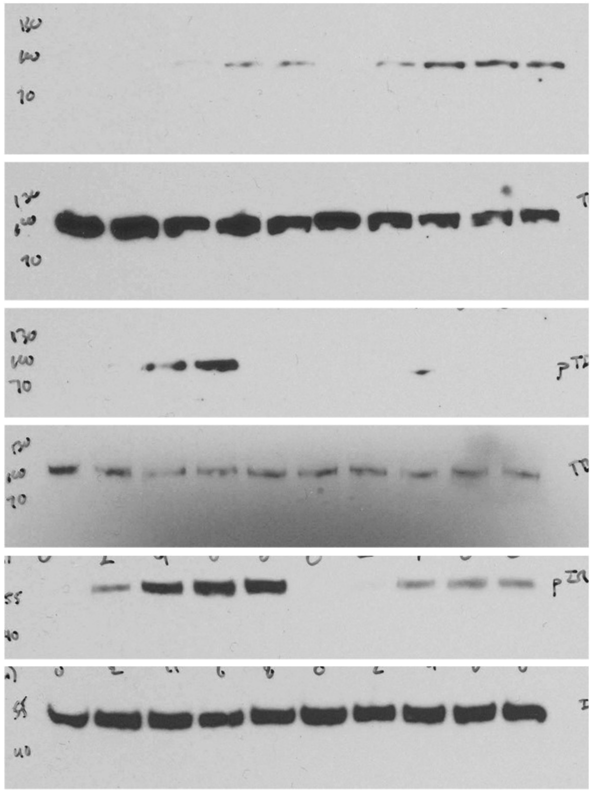

Supplementary Figure 1D

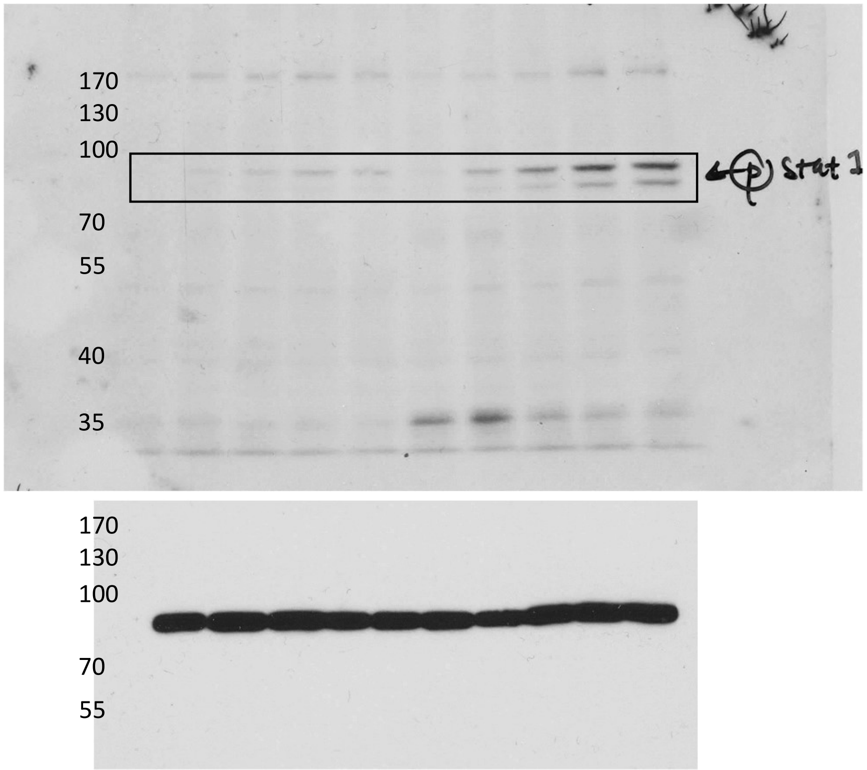

Parvatiyar et al
Supplementary Figure 8

Original Immunoblots Supplementary Figure 1 Continued

Supplementary Figure 1F

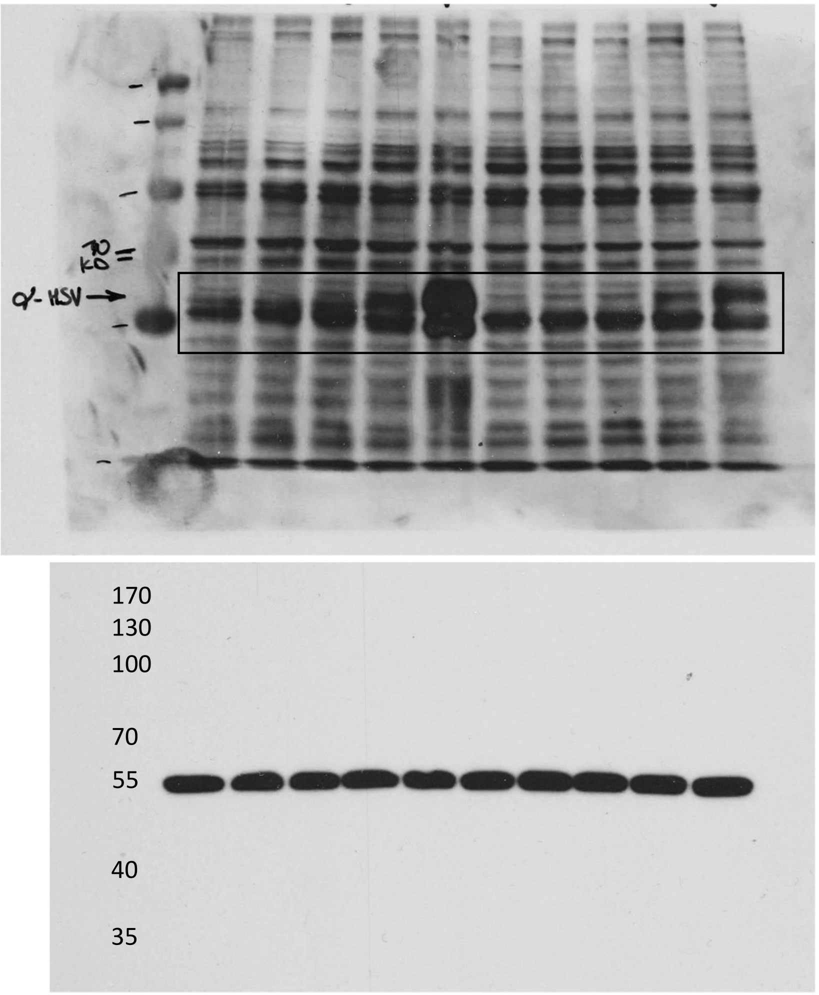

Supplementary Figure 1G

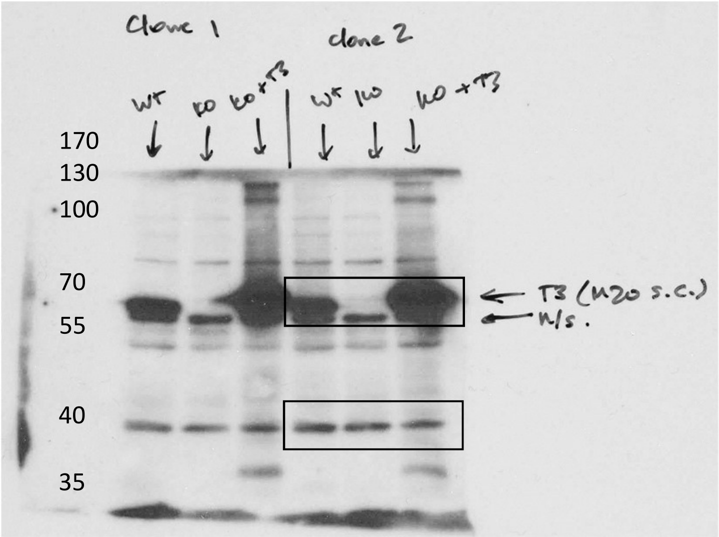

Supplementary Figure 1P

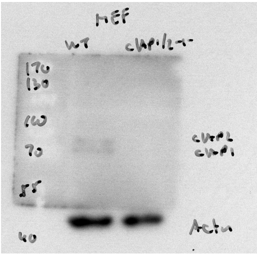

Supplementary Figure 1J

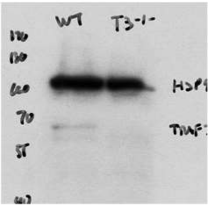

Supplementary Figure 1L

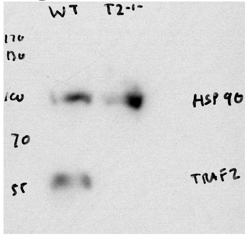

Supplementary Figure 1O

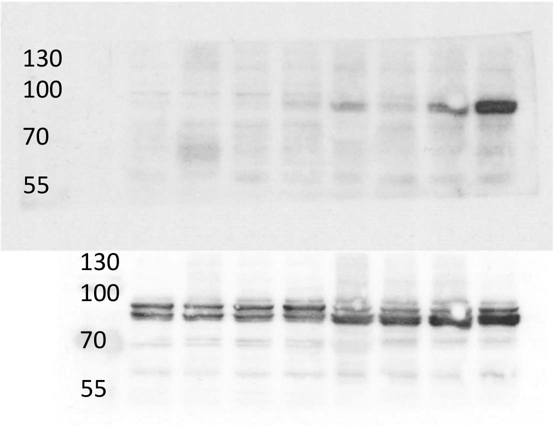

Parvatiyar et al
Supplementary Figure 8

Original Immunoblots Supplementary Figure 2 and 3

Supplementary Figure 2A

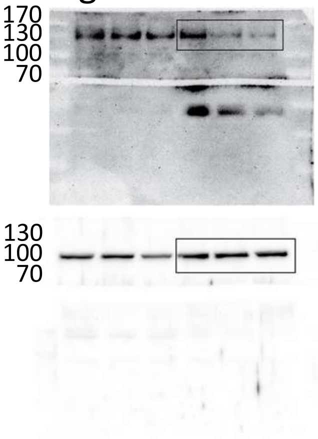

Supplementary Figure 2B

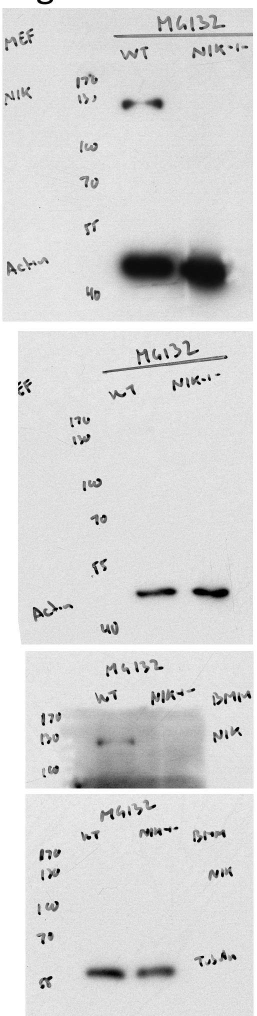

Supplementary Figure 2H

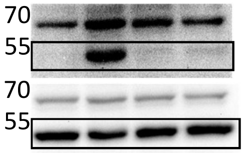

Supplementary Figure 2I

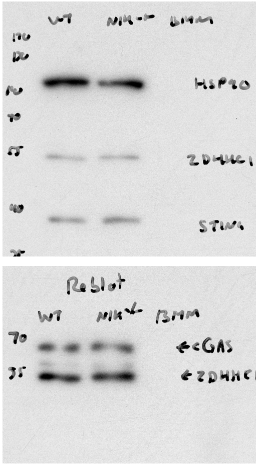

Supplementary Figure 2J

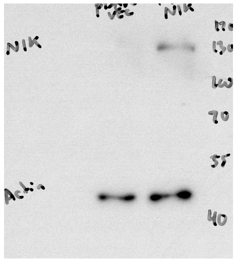

Supplementary Figure 3C

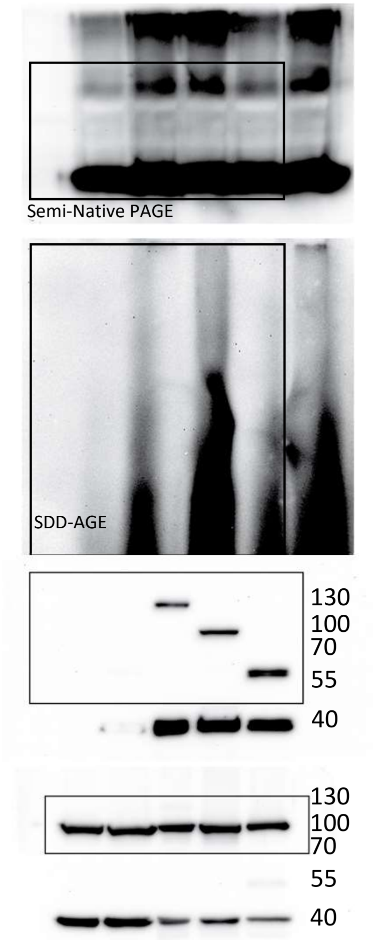

Parvatiyar et al
Supplementary Figure 8

Original Immunoblots Supplementary Figure 4

Supplementary Figure 4A

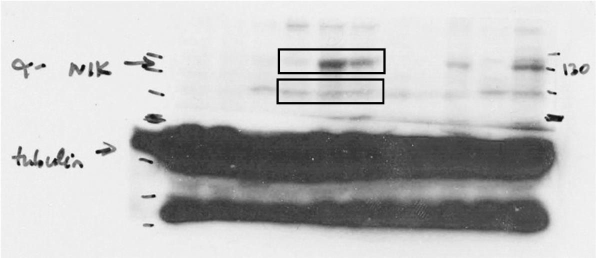

Supplementary Figure 4G

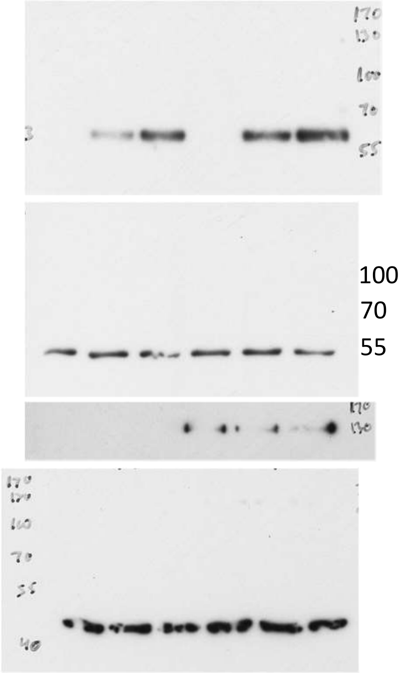

Supplementary Figure 4H

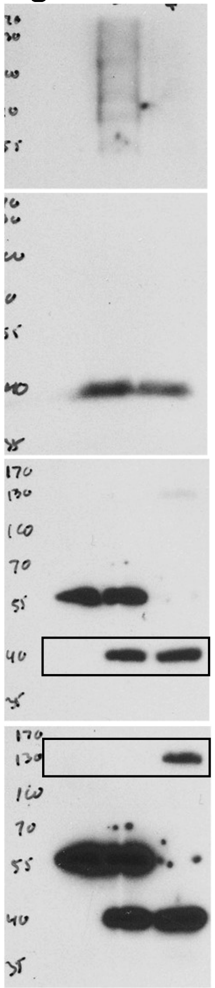

Supplementary Figure 4I

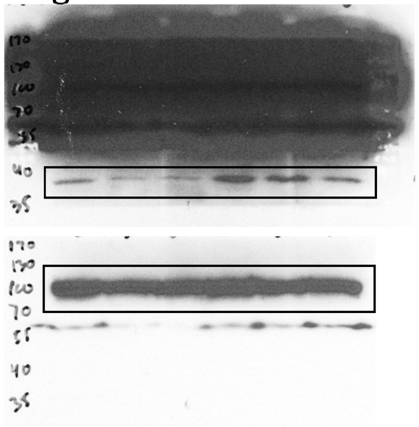

Parvatiyar et al
Supplementary Figure 8

Original Immunoblots Supplementary Figure 5 and 6

Supplementary Figure 5A

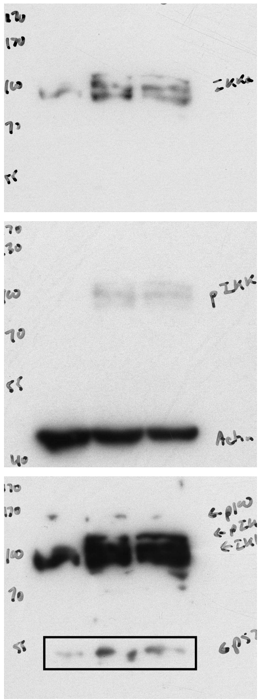

Supplementary Figure 5B

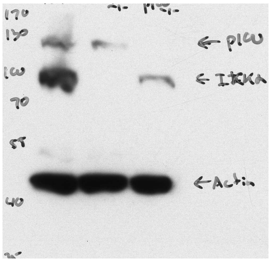

Supplementary Figure 5H

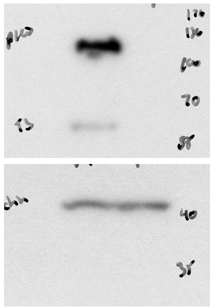

Supplementary Figure 5I

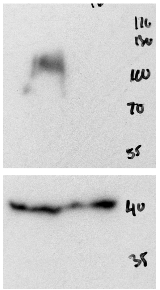

Supplementary Figure 6B

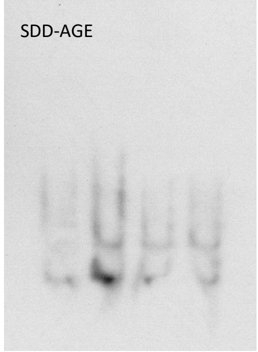

Supplementary Figure 6C

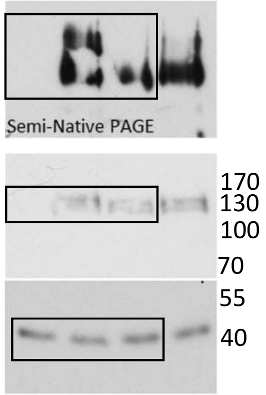

Supplementary Figure 6D

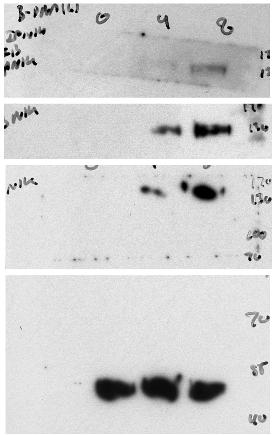

Parvatiyar et al
Supplementary Figure 8
